# Visible-Light-Driven Photocatalytic H_2_ Production Using Composites of Co–Al Layered Double Hydroxides and Graphene Derivatives

**DOI:** 10.1021/acs.inorgchem.4c00671

**Published:** 2024-05-28

**Authors:** Dolores G. Gil-Gavilán, Juan Amaro-Gahete, Daniel Cosano, Miguel Castillo-Rodríguez, Gustavo de Miguel, Dolores Esquivel, José R. Ruiz, Francisco J. Romero-Salguero

**Affiliations:** † Departamento de Química Orgánica, Instituto Químico para la Energía y el Medioambiente (IQUEMA), Facultad de Ciencias, Universidad de Córdoba, Campus de Rabanales, Edificio Marie Curie, 14071 Córdoba, Spain; ‡ UGR-Carbon − Materiales Polifuncionales Basados en Carbono, Departamento de Química Inorgánica, Unidad de Excelencia Química Aplicada a Biomedicina y Medioambiente, Universidad de Granada, 18071 Granada, Spain; § Departamento de Física Aplicada, Radiología y Medicina Física, Universidad de Córdoba, Campus de Rabanales, 14071 Córdoba, Spain; ⊥ Departamento de Química Física y Termodinámica Aplicada, Instituto Químico para la Energía y el Medioambiente (IQUEMA), Facultad de Ciencias, Universidad de Córdoba, Campus de Rabanales, Edificio Marie Curie, 14071 Córdoba, Spain

## Abstract

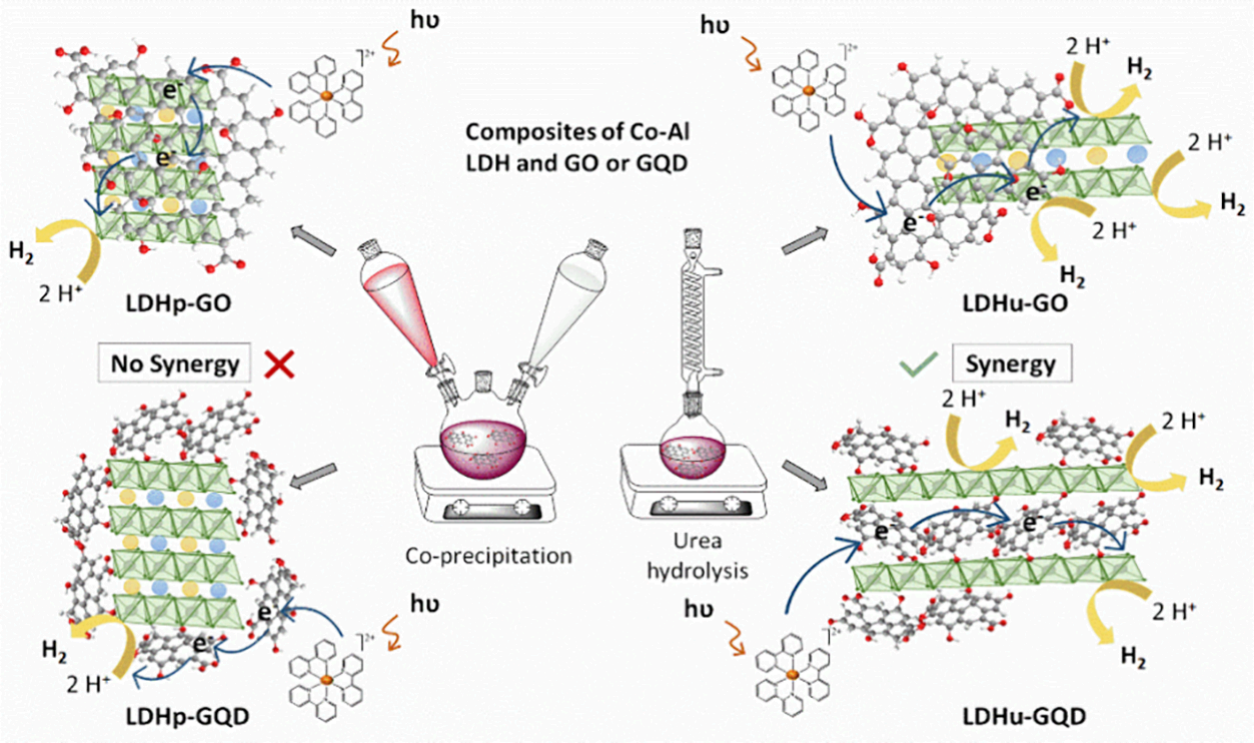

The direct conversion of solar energy into chemical energy represents an enormous challenge for current science. One of the commonly proposed photocatalytic systems is composed of a photosensitizer (PS) and a catalyst, together with a sacrificial electron donor (ED) when only the reduction of protons to H_2_ is addressed. Layered double hydroxides (LDH) have emerged as effective catalysts. Herein, two Co–Al LDH and their composites with graphene oxide (GO) or graphene quantum dots (GQD) have been prepared by coprecipitation and urea hydrolysis, which determined their structure and so their catalytic performance, giving H_2_ productions between 1409 and 8643 μmol g^–1^ using a ruthenium complex as PS and triethanolamine as ED at 450 nm. The influence of different factors, including the integration of both components, on their catalytic behavior, has been studied. The proper arrangement between the particles of both components seems to be the determining factor for achieving a synergistic interaction between LDH and GO or GQD. The novel Co–Al LDH composite with intercalated GQD achieved an outstanding catalytic efficiency (8643 μmol H_2_ g^–1^) and exhibited excellent reusability after 3 reaction cycles, thus representing an optimal integration between graphene materials and Co–Al LDH for visible light driven H_2_ photocatalytic production.

## Abstract 2

Several composites of Co−Al LDH and graphene derivatives have been used as catalysts for visible-light-driven H_2_ production. The factors that allow a proper integration of both components to manifest in a synergistic effect, enhancing the activity of pristine LDH, have been investigated.

## 1 Introduction

Nowadays, there is an urgent need to replace nonrenewable energy resources, such as coal and oil, with alternative fuels that help mitigate climate change and pollution. Hydrogen is an ideal candidate because it acts as a clean energy carrier with high energy conversion and easy regeneration. Obviously, hydrogen production must be carried out with the help of renewable energies such as solar and wind power. In this context, photocatalytic hydrogen production by visible radiation is one of the hot topics of current research.^1−3^

Several types of materials can act as catalysts in systems for the photocatalytic hydrogen production by water splitting, including layered double hydroxides (LDHs). These consist of an inorganic brucite-like laminar structure with octahedral geometry represented by the general formula [M_1–*x*_^II^ M_*x*_^III^ (OH)_2_][A^*n*–^]_*x*/*n*_·*m*H_2_O, where M^II^ and M^III^ are divalent and trivalent cations, respectively, such as Co^2+^, Mg^2+^, Zn^2+^, Ni^2+^, Al^3+^, Cr^3+^, and Fe^3+^, whose molar ratio M^III^/(M^II^+M^III^) is in the range 0.20–0.33.^4−6^ Different anions (A^*n*–^), such as Cl^–^, NO_3_^–^, and CO_3_^2–^, are incorporated in the interlaminar space to counterbalance positive charges.

The great compositional flexibility of LDH in terms of metals, anions, and metal molar ratio makes them promising candidates for photocatalytic hydrogen production.^7^ In addition, the layered structure of LDH materials provides flexibility to accommodate various crystal sizes, shapes, and morphologies. These attributes significantly influence the efficiency of charge transfer and separation, thus determining the performance in photocatalytic energy conversion.^8^ Considering their drawbacks, i.e., poor light absorption in the visible region and fast electron–hole recombination, different investigations have been pursued for the formation of composites with other materials in order to improve their catalytic performance in hydrogen production.^9^

Due to their surface characteristics, graphene oxides (GOs) and graphene quantum dots (GQDs) are particularly interesting for the formation of composites with other materials.^10^ GOs are two-dimensional materials composed of several aromatized sp^2^ layers,^11^ whereas GQDs are zero-dimensional carbon nanomaterials that are less than 15 nm wide and 0.5–2.0 nm thick.^12^ Both are functionalized with oxygen-containing organic functions, such as carboxylic acid, hydroxyl, and epoxide groups.^13^ These graphene-based materials have been widely used in energy and environmental applications.^14^

Composites based on LDH and graphene materials have also been studied because the integration of these two components provides an unique structure that possesses the characteristics and advantages of both starting materials.^15^ Thus, Wang et al. studied the synergistic effect between Co–Al LDH and reduced graphene oxide (rGO) and synthesized an outstanding electrocatalyst for oxygen reduction reaction (ORR) by coprecipitation and subsequent hydrothermal treatment. This research claimed that the synergistic effect was based on the fact that the composite showed higher conductivity as the amount of rGO increased, although an optimal ratio between rGO and LDH was reported.^16^ On the other hand, concerning LDH/GQD materials, a composite integrating N-doped GQD and Ni–Fe LDH synthesized by a hydrothermal method was found to catalyze OER with excellent results.^17^

LDHs usually absorb light very weakly in the visible region, so they are rarely useful on their own as photocatalysts. Indeed, the formation of heterojunctions between LDHs and other materials has been commonly carried out to obtain composites with improved photocatalytic hydrogen production,^18^ being Z-scheme, S-scheme, and type II heterojunctions the most frequently studied. Sun et al. developed a LDH Z-scheme system for the photocatalytic H_2_ production reaction in which the electron transfer occurred from the conduction band (CB) of Zn_0.5_Cd_0.5_S with a more positive potential to the valence band (VB) of the LDH, being the proton reduction carried out in the former band.^19^ Nayak and Parida obtained a Ni–Fe LDH/N-rGO/g-C_3_N_4_ hybrid by calcination, electrostatic self-assembly, and several hydrothermal steps and tested it in photocatalytic hydrogen production with outstanding results. N-rGO acted as electron mediator in a Z-scheme mechanistic route between Ni–Fe LDH and g-C_3_N_4_.^20^ For LDH S-scheme, Li et al. proposed a heterojunction formed by CdSe and LDH. CdSe has a higher Fermi energy level than Co–Al LDH, so the electrons diffused from CdSe to LDH. The alignment of their Fermi energy levels favored the recombination of electrons in the CB of LDH with holes in the VB of CdSe, whereas protons were reduced to H_2_ by electrons in the CB of CdSe.^21^ However, in the type II heterojunction synthesized by Guo et al., the electron transfer is from the CB of the semiconductor with a more negative potential (LDH) to the CB of the second semiconductor (CeO_2_), where protons are reduced.^22^ Sometimes, the addition of a PS is necessary to improve light absorption on heterostructures, as in the case of the S-scheme formed by Ni–Fe LDH and ZIF-67, where Eosin Y was added.^23^ Also, a Co–Al LDH/rGO composite obtained by a solvothermal method exhibited higher catalytic activity than pristine Co–Al LDH using a ruthenium complex as photosensitizer.^24^ Depending on whether the charge-separated excited state of the PS is oxidized or reduced through electron transfer assisted by an acceptor or donor agent, the mechanism could occur by an oxidative^25^ or a reductive quenching, respectively.^23^

Due to its properties and exceptional redox activity, Co–Al LDH is an ideal candidate for photocatalytic hydrogen production.^21^ Furthermore, the formation of composites based of Co–Al LDH and other compounds, such as GO, seems to decrease the electron–hole recombination rate and improve the electronic conductivity.^26^ For these reasons, Co–Al LDH has been used in this work in combination with GO and GQD obtaining composites for H_2_ production.

Herein, different composites consisting of Co–Al LDH and a carbon material, specifically GO or GQD, have been synthesized by coprecipitation and urea hydrolysis approaches. GO and GQD were previously obtained by the Hummers and the citric acid pyrolysis methods, respectively. Composites and pristine Co–Al LDH have been tested as catalysts in photocatalytic systems for hydrogen production under visible light. Different factors affecting the synergy between the two components, i.e., Co–Al LDH and GO or GQD, have been analyzed. To date, the application of a Co–Al LDH with GQD intercalated between layers in the photocatalytic production of H_2_ under visible light has not been previously reported.

## 2 Results and Discussion

### 2.1 Characterization of the Materials

Zeta potential values for GO and GQD at pH = 10 were −27 and −15 mV, respectively, and so both carbon materials can undergo an electrostatic interaction with LDH layers, which are positively charged. Following two synthetic procedures, i.e., coprecipitation and urea hydrolysis, two LDH composites with GO and two with GQD were obtained. As expected, X-ray fluorescence revealed that the Co^2+^ to Al^3+^ ratios for all materials were close to 3 (Table S1). The carbon content of the composites was determined by elemental analysis (Table S1), and the results indicated that those composites obtained by coprecipitation, LDHp-GO and LDHp-GQD, showed similar carbon content (ca. 5 wt %), whereas those synthesized by urea hydrolysis, LDHu-GO and LDHu-GQD, displayed a higher carbon content, particularly LDHu-GQD, which had around 12 wt %, twice that of LDHu-GO.

**Figure 1:**
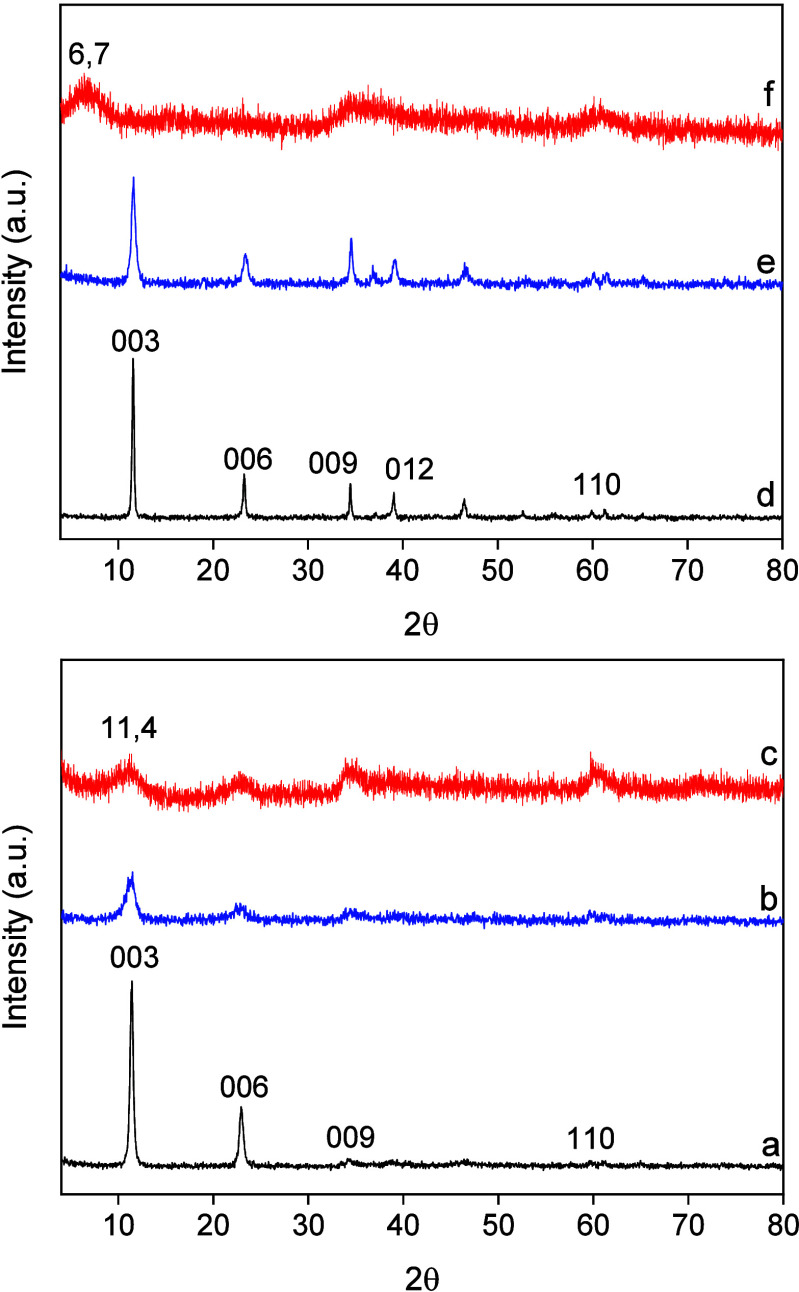
XRD patterns of synthesized materials: (a) LDHp, (b) LDHp-GO, (c) LDHp-GQD, (d) LDHu, (e) LDHu-GO, and (f) LDHu-GQD.

XRD patterns of the starting graphite and GO (Figure S1) exhibited the signals attributed to their characteristic diffraction planes (26.6 and 54.7° for graphite; 11.4 and 42.4° for GO).^27^ The XRD patterns for LDH and composites are shown in Figure 1 and revealed the main diffraction peaks of Co–Al LDH structures (JCPDS: 51–0045).^28^ The *d*_003_ values in all synthesized materials were between 7.6 and 7.8 Å (Table 1), which was indicative of the presence of both carbonate and nitrate as interlaminar anions (*vide infra*).^29^ LDHu exhibited a higher crystallinity than LDHp, as usually observed when comparing both synthesis procedures.^30^ All diffractions signals were much weaker for all composites due to a decrease in crystallinity^28^ with the same trend relative to the synthesis procedure. In addition, those composites with GQD were less crystalline than those with GO. In general, the decrease in crystallinity could be due to the restricted growth of crystallites caused by the electrostatic interactions between LDH layers and graphene-based materials. Interestingly, composite LDHu-GQD presented a basal reflection shifted to a lower angle (Figure 1f), with *d*_003_ and *c* values of 13.2 and 36.9 Å, respectively, indicating a larger interlayer spacing compared to LDHu (Table 1).^31^ The reflections in the rest of the composites were typical of the LDH structure. Accordingly, GQD particles were indeed intercalated between LDH layers in composite LDHu-GQD.

**Table 1:** Lattice Parameters (*d*_003_, *a*, and *c* Values), Crystallite Size (*D*), Crystallinity, Specific Surface Area, and Pore Volume of Synthesized Materials

Material	*d*_003_ (Å)^a^	*c* (Å)^a^	*a* (Å)^a^	*D* (Å)^b^	Crystallinity (%)^c^	*S*_BET_ (m^2^ g^–1^)^d^	*V*_p_ (cm^3^ g^–1^)^e^	*D*[4,3] (μm)^f^
LDHp	7.8	23.3	3.1	179.8	68	34.0 ± 2.0	0.204 ± 0.011	11.1 ± 0.9
LDHp-GO	7.7	23.3	3.1	62.4	53	1.0 ± 0.3	0.005 ± 0.001	86.8 ± 1.3
LDHp-GQD	7.8	23.4	3.1	23.9	28	2.0 ± 0.5	0.002 ± 0.001	89.6 ± 0.9
LDHu	7.6	22.9	3.1	312.6	100	17.0 ± 0.3	0.086 ± 0.019	7.8 ± 0.6
LDHu-GO	7.6	22.9	3.1	148.6	59	30.0 ± 1.0	0.147 ± 0.001	21.6 ± 1.2
LDHu-GQD	13.2	36.9	3.1	18.3	16	3.0 ± 0.5	0.016 ± 0.001	8.3 ± 0.5

a Lattice parameters.

b Crystallite size.

c Relative to LDHu.

d BET surface area.

e Pore volume.

f Volume moment values.

Thermogravimetric analysis (TGA) curves for all synthesized materials exhibited weight loss regions typical of LDH-based materials, i.e., elimination of adsorbed (at ca. 50 °C) and interlayer (close to 200 °C) water molecules (Table S1) and subsequent losses related to dehydroxylation and anion decomposition, which occurred at similar temperatures (250–300 °C) in all materials except for GQD composites (350–400 °C) (Figure S2).^32,33^ The decomposition of graphene derivatives in composites was observed above 550 °C, with a lower temperature for those with GQD than with GO.

Raman spectra revealed the main bands in graphite and graphene derivatives, i.e., the D and G bands, attributed to sp^3^ and sp^2^ carbon domains, which appeared at 1348 and 1577–1595 cm^–1^, respectively (Figure S3).^34^ A signal corresponding to the overtone of the D band, named 2D, appeared at 2701 and 2710 cm^–1^ for GO and graphite, respectively,^35^ whereas an additional band at 2928 cm^–1^ (S3 band) in GO originated from the D–G peak combination.^36^ Also, D and G bands were observed in LDH composites,^24^ and the intensity ratios between these bands (*I*_D_/*I*_G_), which are related to the disorder of graphene materials, were similar (Figure S4). The band occurring at 1041 cm^–1^ in LDHp, synthesized by coprecipitation, corresponded to N–O stretching of nitrate ions (Figure S3c). Pristine material obtained by urea hydrolysis, LDHu (Figure S3d), had a weaker band at 1041 cm^–1^ and a stronger one at 1057 cm^–1^, the latter attributed to C–O stretching of carbonate ions.^37^ The latter was also present in LDHp as a small shoulder. Both materials, as well as the composites, exhibited two bands at 660–695 and 522 cm^–1^ assigned to the F1 2g mode of oxidized cobalt in Co–O bonds and a weaker band at 470–480 cm^–1^ attributed to Al–OH symmetric stretching.^38,39^ FTIR-ATR spectra (Figures S5 and S6) confirmed all these findings.

Textural properties were studied by N_2_ adsorption–desorption isotherms (Figures S7 and S8). Specific surface area and pore volume are summarized in Table 1. LDH-based materials mostly exhibited type II isotherms, characteristic of nonporous or macroporous layered double hydroxides (Figure S8).^40^ Those composites obtained by coprecipitation displayed very low specific surface area and pore volume, suggesting massive pore blocking due to surface deposition of the graphene derivatives. This trend was also shown for LDHu-GQD, whose higher interlayer spacing suggested the decoration of LDH layers by GQD, thus explaining its low surface area. Higher surface area and pore volume were obtained for LDHu-GO, suggesting less blockage of LDH layers and higher contribution of GO for adsorption. In general, better textural properties could be beneficial for photocatalytic reactions.^41^

The morphological characterization of materials was performed by scanning electron microscopy (SEM) and high-resolution transmission electron microscopy (HRTEM). As shown in SEM micrographs, platelet particles forming agglomerates were observed, larger in LDHu than in LDHp (Figure S9). Composites presented a similar morphology with smaller particle sizes (Figures S10 and S11).

Figure S12a shows the HRTEM microstructure of GO, which was composed of several transparent sheets, normally wrinkled at the edges as indicated in the bottom right inset.^42^ HRTEM performed at the edge revealed a crystalline structure whose fast Fourier transform (FFT) analysis provided *d* space values of about 0.38 and 0.21 nm. They corresponded to the GO sheets distance and the *d*_100_ interplanar space for GO, respectively.^43^ Several GQDs are depicted in Figure S12b. They were about 5–10 nm in diameter, and the FFT pattern indicated a spacing of 0.21 nm that could be ascribed to (100) planes.^44^

Typical hexagonal nanosheets were observed in pristine LDH synthesized by coprecipitation (LDHp) using TEM (Figure S13a), where (012) and (110) planes were identified in their corresponding selected area electron diffraction (SAED) patterns. EDS elemental mapping analysis confirmed the presence of Co and Al in a 3:1 atomic % ratio (Figure S13b). The microstructure of pristine LDH synthesized by urea homogeneous precipitation (LDHu) is shown in Figure S14a. It is worth emphasizing that hexagonal sheets were much larger with sizes in the micrometric range. Crystallinity was preserved, as attested by SAED patterns, and chemical composition also exhibited a 3:1 Co:Al atomic % ratio (Figure S14b).

**Figure 2:**
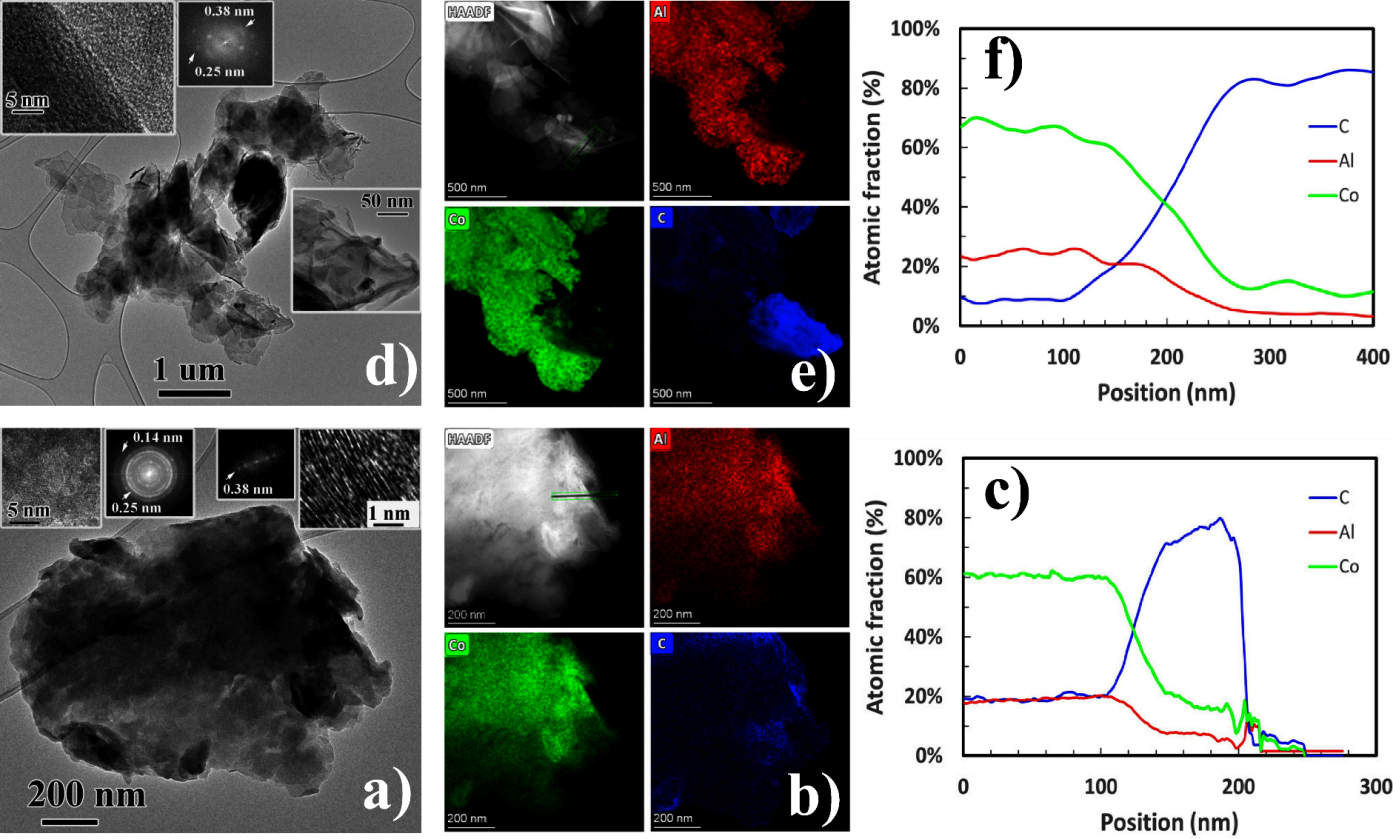
(a) LDHp-GO composite. FFT pattern of the magnified upper left inset is composed by rings with 0.25 and 0.14 nm ascribed to (012) and (110) LDH planes, respectively. The upper right inset corresponds to GO sheets as confirmed by FFT. (b) EDS elemental mapping and (c) atomic fraction profile (marked by a green arrow in HAADF image) clearly indicating a Co:Al ratio very close to 3:1. The rise on C content should be due to the GO presence. (d) LDHu-GO composite. Right inset is showing several wrinkled GO layers. The FFT of the upper left inset shows the contribution coming from both, LDHu and GO materials. (e) EDS elemental mapping and (f) atomic fraction profile confirming again a 3:1 ratio for Co:Al elements.

In order to distinguish between both components in composite LDHp-GO, HRTEM and FFT analysis were carried out (Figure 2a). Upper left inset corresponds to LDH layers where FFT pattern was comprised by rings due to the high number of contributing LDH layers. It was possible to identify rings with 0.25 and 0.14 nm ascribed to (012) and (110) planes, respectively. However, upper right inset shows lattice fringes of GO as confirmed by FFT analysis. EDS elemental mapping performed close to the edge is shown in Figure 2b. An atomic concentration profile had been performed where the increase in C concentration was really evident (Figure 2c). Moreover, Co:Al ratio was found to be 3:1 as was corroborated by XRF.

The microstructure of LDHu-GO (Figure 2d) composite was very similar, but LDH sheets synthesized by urea homogeneous precipitation were much larger, and moreover, very few layers were contributing to FFT pattern. In consequence, no rings but individual spots were observed. The (012) plane with a *d* value of 0.25 nm coming from LDHu and the GO sheets distance of 0.38 nm were identified. EDS elemental maps also indicated the presence of areas with higher carbon concentration ascribed to GO layers (Figure 2e). The rise of the C concentration and the Co:Al ratio of about 3:1 have been observed along the atomic concentration profile shown in Figure 2f.

**Figure 3:**
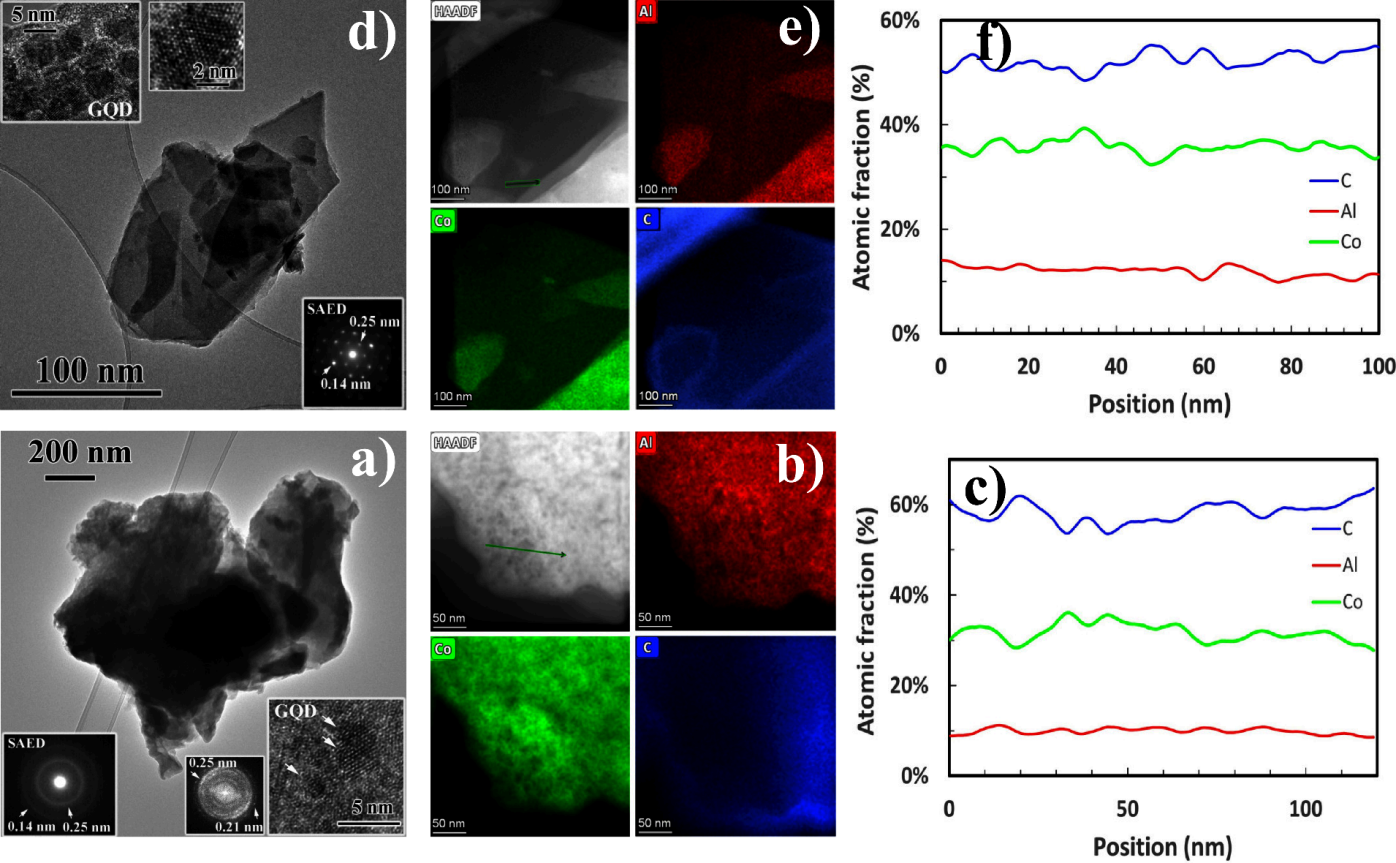
(a) LDHp-GQD composite. Lower left inset displays the SAED pattern obtained from the composite, where intensities from GQD contribution is so weak that is not visible. Lower right inset is a HRTEM image of few GQD. FFT is also shown. (b) EDS elemental mapping and (c) atomic fraction profile where it is observed the 3:1 ratio for Co:Al elements and fluctuations in C content due to the presence of GQD along the profile. (d) LDHu-GQD composites. Lower right inset displays the SAED pattern composed exclusively by LDH spots due to the weak GQD contribution. Upper left insets show several GQD. (e) EDS elemental mapping and (f) atomic fraction profile confirming again the 3:1 ratio for Co:Al elements and fluctuations in C content due to the presence of GQD along the profile.

Regarding LDHp-GQD and LDHu-GQD microstructures (Figure 3), both were very similar, although again LDHu-GQD exhibited larger sheets and a lower number of stacked layers than LDHp-GQD, as evidenced in the micrographs and the rings and individual spots found for LDHp-GQD and LDHu-GQD in the SAED pattern, respectively. (012) and (110) planes with *d* values of 0.25 and 0.14 nm coming from LDH layers were identified, although contributions to these SAED patterns coming from GQD were not observed due to the very low GQD:LDH electron beam scattered volume ratio. As a consequence, GQD intensities were very weak compared to layered double hydroxide ones, and therefore, they were not visible. Nevertheless, GQD were clearly observed by HRTEM, as displayed in the insets of Figure 3a and d, and also by EDS elemental maps (Figure 3b,e). Thus, the atomic fraction profile performed for both samples, LDHp-GQD and LDHu-GQD composites (Figure 3c,f), besides confirming a Co:Al ratio very close to 3:1, also showed fluctuations in C at. % content. These fluctuations were not observed in samples with no GQD content (Figure 2). Moreover, these fluctuations were about 5 and 10 nm in width, which were in very good agreement with GQD width measurements obtained by HRTEM (Figure S12b).

**Figure 4:**
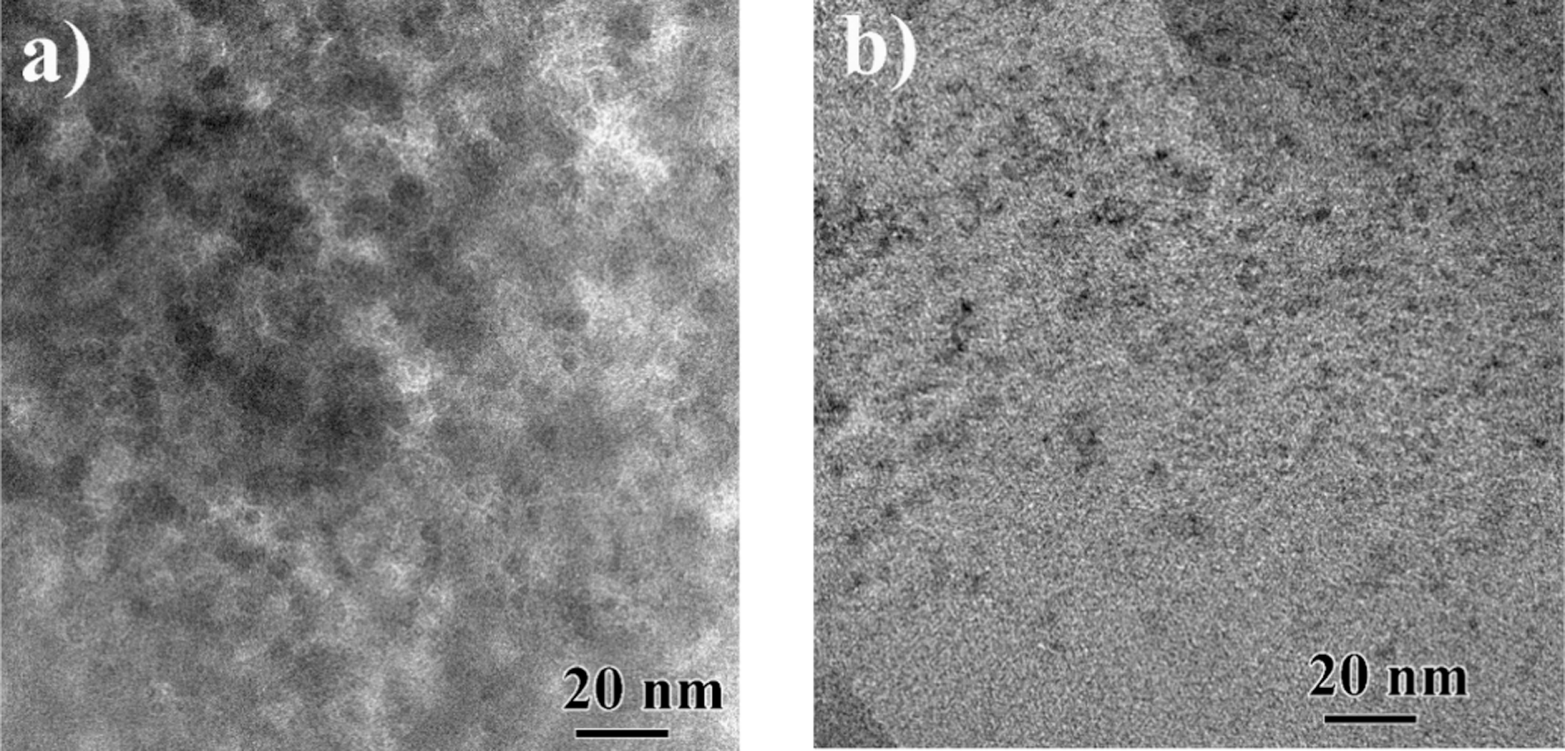
TEM micrographs for (a) LDHp-GQD and (b) LDHu-GQD composites. To enhance the contrast of GQD, an intermediate size objective aperture was inserted so that GQDs get darker in the micrographs.

Related to the integration of both components in the composites, i.e., LDH and GO or GQD, additional TEM studies were conducted. Many areas were investigated to check whether the synthesis process, coprecipitation, or urea homogeneous precipitation had any influence on the GQD distribution throughout the LDH layers (Figure 4). For LDHp-GQD composite, it was observed that GQDs were quite agglomerated, covering the LDH surface completely. By contrast, LDHu-GQD exhibited GQDs reasonably well distributed throughout the surface, leaving some free area.

Particle size distributions for pristine materials and composites were obtained by TEM (Figures S15 and S16). As shown in Figure S15, most particles in GQD were between 2.9 and 4.4 nm, whereas particle size in GO ranged between 200 and 500 nm (Figure S12). Main particle sizes for LDHp and LDHu were in the size ranges of 135–198 nm and 1088–1428 nm, respectively (Figure S16). For coprecipitation synthesized composites, the main LDH particle sizes were 43–51 nm and 51–58 nm for LDHp-GQD and LDHp-GO, respectively, whereas for urea hydrolysis synthesized composites, they were 70–89 nm and 252–293 nm for LDHu-GQD and LDHu-GO, respectively (Figure S16). For the latter composites, a greater decrease in main particle size was observed with respect to LDHu than in coprecipitation synthesized composites.

Volume moment mean values, *D*[4,3], measured by laser diffraction, are given in Table 1. GO displayed a *D*[4,3] of 11.2 μm. In general, LDH composites synthesized by coprecipitation exhibited larger particle sizes than the corresponding materials obtained by urea hydrolysis. Taking into account that crystallite size and main particle size of LDH determined by TEM in materials prepared by coprecipitation were smaller (*vide supra*), these results from laser diffraction indicated that they were more agglomerated in water. In fact, it was clearly observed that these materials displayed lower dispersibility, which may be detrimental to the photocatalytic activity.

UV–vis diffuse reflectance spectra are shown in Figures S17 and S18. Co–Al LDH-based materials exhibited different absorption features, i.e., a broad band centered at 530 nm corresponding to d–d transitions of Co^2+^ (d^5^) in octahedral coordination by weak-field ligands,^45^ a weak broad band at ca. 650 nm due to spin–orbit coupling,^41^ and a band around 260 nm attributed to a ligand to metal charge transfer transition (Figure S18). No absorption bands associated with Al^3+^ were observed due to its d^0^ configuration.^46^ The calculated band gaps were 2.2, 2.1, 2.1, and 2.1 eV for LDHp, LDHp-GQD, LDHu, and LDHu-GQD, respectively (Figure S18).^41^ The band gaps for composites with GO were difficult to obtain accurately due to their broad absorption peaks with an equivocal absorption edge in the UV–vis diffuse reflectance spectra.

### 2.2 Photocatalytic H_2_ Production

Photocatalytic tests for hydrogen production under visible light (λ= 450 nm) were carried out in three-component systems consisting of the synthesized materials as catalysts, Ru(bpy)_3_^2+^ as photosensitizer (PS) and TEOA as sacrificial electron donor (ED). Previously, control tests were performed in absence of catalyst, ED or PS. The two latter components were essential for the reaction, and so zero conversion was obtained in such cases. However, a small amount of H_2_ (744 μmol H_2_) was achieved in the absence of catalyst, which was attributable to the sensitizer Ru(bpy)_3_^2+^, as reported previously.^25^ Moreover, GO and GQD were inactive because the H_2_ produced, 652 and 601 μmol H_2_ g^–1^, respectively, can be ascribed to the sensitizer.

**Figure 5:**
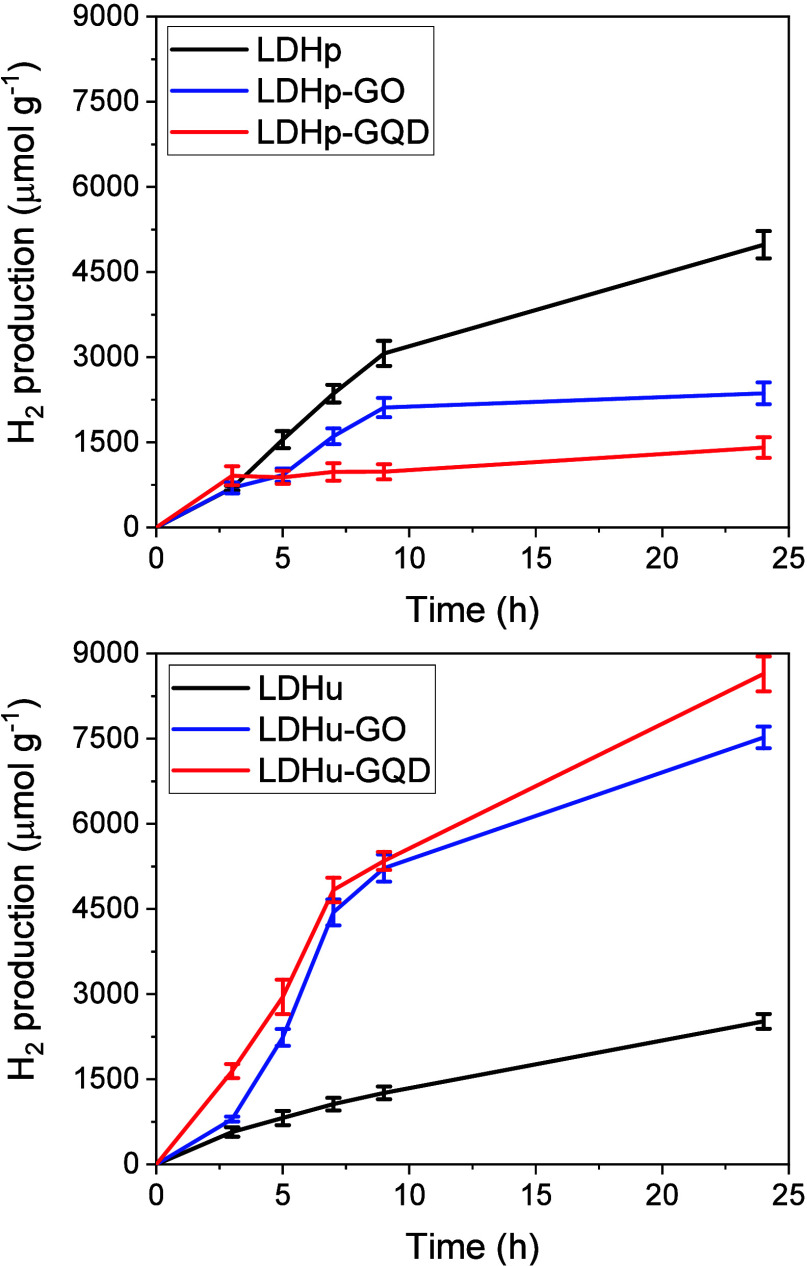
Hydrogen production for the photocatalytic systems.

Composites and layered double hydroxides were active in the reaction. As expected, LDHp exhibited much higher catalytic activity than LDHu (Figure 5). The best textural properties and the smaller particle size shown by LDHp would explain its improved performance. When those materials obtained by coprecipitation were compared, it was observed that the two composites, i.e., LDHp-GO and LDHp-GQD, showed much lower hydrogen production than LDHp. Thus, the H_2_ production at 24 h was 4981, 2364, and 1409 μmol H_2_ g^–1^ for LDHp, LDHp-GO, and LDHp-GQD, respectively. A very marked deterioration of the textural properties of composites with respect to LDHp was observed (Table 1). Besides a drastic decrease in surface area and pore volume, both composites were much more agglomerated in water, which could explain their lower photocatalytic performance.

The case of those materials obtained by urea hydrolysis was quite different. Composite LDHu-GO had larger specific surface area and pore volume than LDHu, but that was not the case for composite LDHu-GQD, which had very low surface area and pore volume. The latter two also showed similar *D*[4,3] values, while LDHu-GO gave the largest particle sizes among these materials. However, both composites showed a large increase in H_2_ production, about 3-times higher than that of LDHu (Figure 5). Thus, LDHu, LDHu-GO, and LDHu-GQD gave 2521, 7523, and 8643 μmol H_2_ g^–1^, respectively, after 24 h. These catalytic systems were quite stable, and their activities did not reach a plateau at short reaction times, in contrast to other systems reported in the literature.^24^ However, production rate decreased with time due to Ru(bpy)_3_^2+^ photodegradation.^47^ Composites LDHu-GO and LDHu-GQD outperformed the H_2_ production obtained with a highly active composite based on Co–Al LDH and carbon spheres (6643 μmol H_2_ g^–1^), previously reported by our group, under analogous conditions.^25^

An additional photocatalytic experiment with the physical mixture of LDHu and GO (6.6 wt %) was carried out. Hydrogen production was stable from 3 to 24 h (978 to 1176 μmol H_2_ g^–1^), but the activity of these mixture was lower than that of all LDH materials and composites synthesized. Therefore, the composite formation was essential to enhance photocatalytic hydrogen production.

Values of apparent quantum efficiency (AQE), also named apparent quantum yield (AQY), were calculated for each photocatalytic system at 450 nm for 5 h. The values for the coprecipitation obtained materials were 2.1, 1.2, and 1.2% for LDHp, LDHp-GO, and LDHp-GQD, respectively, whereas for homogeneous precipitation, synthesized materials were 1.1, 3.0, and 4.0% for LDHu, LDHu-GO, and LDHu-GQD, respectively. AQE values confirmed the previously mentioned photocatalytic performance of the proposed systems and are in concordance with other reported results.^48^

Reusability experiments were carried out with LDHu-GO and LDHu-GQD as catalysts for three runs (Figure S19). A small decrease in photocatalytic activity was observed after each run. After three reactions, LDHu-GO and LDHu-GQD preserved 67 and 87% of the initial H_2_ production at 24 h, respectively. XRD and FTIR-ATR measurements of the reused materials (Figure S20) indicated that their structures were not altered after 72 h of irradiation.

**Scheme 1:**
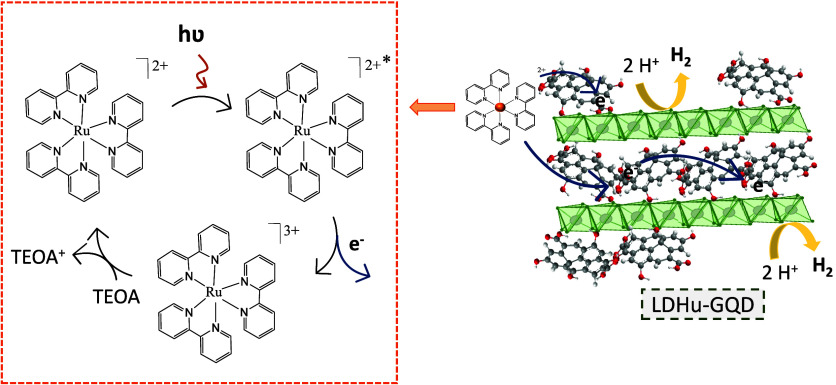
Photocatalytic Mechanism for Light-Driven Hydrogen Production Using Ru(bpy)_3_^2+^, TEOA, and LDHu-GQD as PS, ED, and Catalyst, Respectively

Photoluminescence (PL) experiments were conducted to gain some insights into the mechanism of H_2_ production. PL intensity of the Ru complex upon excitation at 450 nm was not affected by the presence of TEOA, thus ruling out a reductive quenching mechanism.^49^ GO and GQD are known to be strong quenchers for the fluorescence of Ru complexes.^50^ Indeed, a decrease in PL intensity occurred when GO was present (Figure S21). Similarly, composites LDHu-GO and LDHu-GQD produced PL quenching but in different extension, being more intense for LDHu-GO. Additionally, PL quantum yields (PLQYs) were determined correcting the light-scattering caused by the suspended particles.^51^ Thus, the PLQY values obtained for Ru(bpy)_3_^2+^ alone and in the presence of GO, LDHu-GO and LDHu-GQD were 4.89, 2.68, 0.79, and 4.47%, respectively. Given the intense quenching of the PL in the presence of the GO material, it is expected that the visible light irradiation activates an oxidative quenching pathway.^25^ Thus, in a first step, one electron is promoted from the HOMO to the LUMO orbitals in Ru(bpy)_3_^2+^ upon irradiation, generating the excited state (PS*). In a second step, the PS* is oxidatively quenched by electron transfer to the GO or GQD materials and finally to the catalyst, which eventually reduces protons to give H_2_. The role of the PS is essential since H_2_ production is null in its absence. The oxidized PS, i.e., Ru(bpy)_3_^3+^, is reduced back to its initial form, Ru(bpy)_3_^2+^, by the sacrificial electron donor, TEOA, which is oxidized to TEOA^+^ (Scheme 1).^24^

Since electron transfer between the PS and the catalyst must involve a close proximity between both materials, we proceeded to perform adsorption experiments of the PS on LDHu and GO. In the case of GO, the values obtained were fitted to a Langmuir isotherm (Figure S22).^52^ The maximum adsorption capacity obtained was 109 μmol of PS per gram of GO, with an *R*^2^ = 0.98. For LDHu, the adsorption values were negligible, less than 3 μmol of PS per gram of LDH, and could not fit any isotherm. This result was to be expected considering the surface charge of each component of the composite and of PS itself. Certainly, in the case of GO composites, electron transfer appears to occur from the PS to GO, which in turn gives up electrons to the LDH catalyst.

At this point, the question arises as to what role each component of the composite plays in its catalytic performance. It is clear that the combination of GO or GQD with Co–Al LDH influences its catalytic behavior, sometimes positively and occasionally negatively. As discussed above, graphene materials could quench the excited state of the PS without producing the hydrogen evolution reaction. This is observed for materials synthesized by coprecipitation, i.e., LDHp-GO and LDHp-GQD showed lower activity than LDHp. However, this is not the case for those synthesized by urea hydrolysis.

Considering that the presence of the graphene material improves the adsorption of the PS and that, as indicated by other authors,^53^ their high conductivity would facilitate the electronic transfer to LDH, all composites should have improved the catalytic performance with respect to the pristine LDH, through a synergistic effect between both components. In fact, this behavior occurs for composites synthesized by urea hydrolysis but not for those synthesized by coprecipitation.

In short, only with the right choice of the synthesis conditions, which allow a correct integration of the graphene material and the catalyst, in this case LDH, can a synergistic effect between both components be achieved, giving rise to materials with superior catalytic performance.

## 3 Conclusions

In this work, composites of Co–Al LDH and GO or GQD have been obtained by two synthesis procedures, i.e., coprecipitation and urea hydrolysis. All composites had analogous structures (LDH lattice parameters) and composition (Co/Al ratio and carbon content), except LDHu-GQD, which had double carbon content, with GQD particles intercalated between LDH layers. Crystallinity and crystallite size decreased in the composites with respect to the corresponding pristine LDH, being more pronounced in the case of composites with GQD. Their microstructures revealed that the LDH layers in the composites obtained by urea hydrolysis presented a larger size and a smaller number of stacked layers. TEM results indicated that the presence of the graphene materials during the synthesis of the composites decreased the main particle size of LDH. Moreover, the particle size determined by laser diffraction in the composites synthesized by coprecipitation was larger than in those obtained by urea hydrolysis, reflecting a higher agglomeration of LDH and GO or GQD particles.

LDH and composites were active as catalysts in photocatalytic systems with Ru(bpy)_3_^2+^ as sensitizer and TEOA as sacrificial electron donor. A comparison between the pristine LDH activity revealed the importance of textural and morphological properties. However, the incorporation of GO or GQD into the composites can be either positive or negative in relation to their catalytic activity. In fact, a synergistic effect between both components, i.e. LDH and GO or GQD, only occurred when the composites were synthesized by urea hydrolysis. Apparently, textural properties were not decisive in these cases. The graphene material facilitated the adsorption of the PS, which upon light absorption initiated the reaction via an oxidative quenching mechanism. However, both GO and GQD were only able to exert a synergistic effect when properly integrated into the microstructure of the composites, leaving areas of the LDH surface accessible to the reactants. Thus, while for pristine LDHp and LDHu the H_2_ production at 24 h was found to be between 4981 and 2521 μmol g^–1^, respectively, it reached 8643 μmol g^–1^ for the most active composite, LDHu-GQD.

This research improves our knowledge on the design and synthesis of hybrid structures of graphene derivatives and Co–Al LDH with enhanced H_2_-producing photocatalytic activity and remarkable stability. In particular, the correct integration of GQD between Co–Al LDH layers resulted in a material with excellent catalytic performance. These findings could also be of interest for composite formation between other types of materials and GO or GQD.

## 4 Experimental Section

### 4.1 Materials and Reagents

GO was obtained using the following commercial reagents: Graphite powder (Sigma-Aldrich, ref. 282863–1KG), H_2_SO_4_ (PanReac, 95–98%), NaNO_3_ (PanReac), KMnO_4_ (Sigma-Aldrich, 99%), H_2_O_2_ (PanReac, 30%), HCl (GlobalChem, 37%). Monohydrated citric acid (Sigma-Aldrich, 99%) was pyrolyzed for the synthesis of GQD. Co(NO_3_)_2_·6H_2_O (Sigma-Aldrich, 98%), Al(NO_3_)_3_·9H_2_O (Sigma-Aldrich, >98%), NaOH (Panreac, 98%), and urea (PanReac) were used to synthesize LDH and composites. Tris(2,2′-bipyridyl)dichlororuthenium(II) hexahydrate (Sigma-Aldrich, >98%), triethanolamine (TEOA, Sigma-Aldrich, >99%), and acetonitrile (PanReac, 99.7%) were employed in the photocatalytic reactions for hydrogen production.

### 4.2 Characterization Techniques

Zeta potential measurements of graphene-based materials were carried out on a zeta potential analyzer (ZetaSizer Nano ZSP, Malvern). The Co/Al ratio in the LDH and the composites was determined from X-ray fluorescence (XRF) spectroscopy. Spectra were acquired with a Rigaku ZSK Primus IV instrument. Elemental analyses were carried out in a Thermo Scientific Elemental Analyzer CHSN TM FlashSmart. X-ray powder diffraction (XRD) patterns were collected over the 2θ range 3–80° in a Bruker D8 Discover A25 diffractometer using Cu Kα radiation. Lattice parameters were calculated according to the following expressions: *c* = 3/2(*d*_003_ + *d*_006_) and *a* = 2*d*_110_.^54^ Crystallite size was calculated according to the Scherrer equation, *D*_hkl_ = *R*(λ/β cos θ), where *R* is the Scherrer number (0.89), λ is the incident X-ray wavelength (0.154 nm), β is the peak width at half height (rad), and θ is the Bragg angle.^55^ Crystallinity percent of synthesized materials was calculated considering the areas of (003) and (110) reflections. Thermogravimetric analyses (TGAs) were performed using a PerkinElmer TGA8000 equipment in the temperature range of 25–1100 °C, with a heating rate of 5 °C min^–1^ at a N_2_ flow rate of 40 mL min^–1^. Raman spectra of the samples were acquired with a Renishaw Raman instrument with green laser light (532 nm) over the wavenumber range 400–4000 cm^–1^. FTIR-ATR measurements were carried out on a PerkinElmer FRONTIER spectrometer over the wavenumber range 450–4000 cm^–1^. Nitrogen adsorption–desorption isotherms were obtained in a Micromeritics ASAP 2000 system at −196 °C. Samples were outgassed at 70 °C before the measurement. Brunauer–Emmett–Teller (BET) method was used for determining the surface area. Scanning electron micrographs were obtained with a JEOL JSM 7800 microscope at a voltage of 15 kV. TEM and HRTEM were performed using a FEI Talos F200i S/TEM microscope operating at 200 kV. STEM mode using the high angle annular dark field (HAADF) detector was also carried out, providing Z contrast imaging. Moreover, elemental mapping was carried out using energy dispersive X-ray spectroscopy (EDS). Particle size measurements were carried out in a Mastersizer 2000 laser diffraction analyzer (Malvern Instruments) equipped with a Hydro 2000 SM sample dispersion unit. The measurements for each material were repeated 3 times, and the dispersant used was deionized water. Volume moment mean values, *D*[4,3], were acquired in order to gather information about the average size of the particles that constitute the bulk of the sample volume. Ultraviolet–visible (UV–vis) diffuse reflectance spectra were measured by using a Scan UV–vis spectrophotometer (PerkinElmer Lambda 650 S). Band gaps values were calculated using Kubelka–Munk function from the plots of (*F*(*R*) *h*υ)^2^ vs *h*υ. Apparent quantum efficiency was calculated for each photocatalyst using the following equation: AQE = (2 × number of evolved H_2_ molecules/number of incident photons) × 100.^48,56^ Ru(bpy)_3_^2+^ adsorption onto the GO or LDH was assessed by adding different amounts of one of these solids in an CH_3_CN/H_2_O solution of Ru(bpy)_3_^2+^ (11.4 mL, 3.95 × 10^–4^ M). The tests were carried out under similar conditions to the photocatalytic experiments, but the samples were shaken in the darkness for 24 h. The concentration of Ru(bpy)_3_^2+^ after contact with the material was determined in a double-beam UV–vis 4260/50 (ZUZI) instrument in a wavelength range of 250–800 nm. The Langmuir isotherm was used to calculate the amount of adsorbate assuming a homogeneous sorption in a monolayer form. The model can be expressed with the linear form (eq 1):^57^
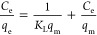
1where *C*_e_ and *q*_e_ are the adsorbate concentration (g L^–1^) and the amount of Ru complex adsorbed at equilibrium (g g^–1^), respectively, *K*_L_ is the Langmuir constant, and *q*_m_ denotes the maximum adsorption capacity (g g^–1^).

### 4.3 Synthesis of Materials

#### 4.3.1 Synthesis of Graphene Oxide

GO was synthesized by a modified Hummers method using graphite as starting material. Graphite (3.0 g) and sodium nitrate (1.5 g) were added to concentrated sulfuric acid (70 mL) under stirring. The mixture was cooled to 0 °C, and potassium permanganate (9.0 g) was added slowly to keep the temperature at 20 °C. After 15 min, the reaction was left to stand at 40 °C for 30 min. Then 140 mL of water was added, and the suspension was stirred for 15 min at 90 °C. H_2_O_2_ was slowly added and, while stirring, the heater was turned off. Then the solid was filtered, washed with 10% HCl and deionized water until the pH of the supernatant was ca. 7. After that, the material was dried overnight at 60 °C and finally ball milled to obtain a powder.^58^

#### 4.3.2 Synthesis of Graphene Quantum Dots

GQDs were synthesized by pyrolysis of 2.0 g of citric acid at 200 °C for 30 min. An orange liquid was obtained.^12^

#### 4.3.3 Synthesis of Pristine LDH

Conventional coprecipitation and urea hydrolysis methods were used to synthesize pristine LDHs. A typical procedure of coprecipitation^59^ consisted of mixing 0.015 mol of Co(NO_3_)_2_·6H_2_O and 0.005 mol of Al(NO_3_)_3_·9H_2_O in 150 mL of deionized water (i.e., Co/Al ratio of 3) and then slowly (2 h) dropping the mixture over 500 mL of deionized water at 60 °C under vigorous stirring. The pH was kept constant (pH = 10) throughout by adding appropriate volumes of 1 M NaOH when needed. The suspension thus obtained was maintained at 80 °C for 24 h, after which it was filtered and washed with 2 L of deionized water to obtain the material named LDHp.

Urea hydrolysis method (also denoted urea homogeneous precipitation) consisted of mixing urea (0.150 mol) with the solution of 0.015 mol of Co(NO_3_)_2_·6H_2_O and 0.005 mol of Al(NO_3_)_3_·9H_2_O in 100 mL of water. The temperature was raised up to 90 °C while stirring for 42 h. Finally, the product was filtered, washed several times with deionized water, and dried in vacuum at 80 °C overnight.^60^ The material obtained was named LDHu.

#### 4.3.4 Synthesis of Composites

The composites were obtained similarly to the previously described methods. Following the coprecipitation method, the mixture of salts, 0.015 mol of Co(NO_3_)_2_·6H_2_O and 0.005 mol of Al(NO_3_)_3_·9H_2_O, was added to either a 500 mL deionized water suspension of 250 mg of GO or a 500 mL deionized water solution of 1.0 g of GQD. The solutions were previously sonicated for 90 min to ensure graphene material dispersion. The addition time was 2 h, and the synthesis was at 60 °C and pH = 10. Two materials named LDHp-GO and LDHp-GQD were obtained. Following the urea homogeneous precipitation method, the mixture of salts, 0.015 mol of Co(NO_3_)_2_·6H_2_O and 0.005 mol of Al(NO_3_)_3_·9H_2_O, and urea (0.150 mol) were added to either a 100 mL deionized water suspension of 250 mg of GO or a 100 mL deionized water solution of 1.0 g of GQD. Two materials denoted LDHu-GO and LDHu-GQD were obtained.

### 4.4 Experimental Conditions of Photocatalytic H_2_ Production

Photocatalytic hydrogen production was studied to investigate the catalytic performance of all prepared materials. A Penn PhD Photoreactor M2 with λ = 450 nm was used as the light source (2524 W/m^2^). The reaction was carried out in a sealed vessel containing 1.30 mg of catalyst dispersed in 11.40 mL of a solution containing 2.85 mL of TEOA (2.14 mmol) solution (0.76 M) in H_2_O and 8.55 mL (3.38 × 10^–3^ mmol) of Ru(bpy)_3_Cl_2_·6H_2_O solution (3.95 × 10^–4^ M) in CH_3_CN. Before irradiation, the vessel was deoxygenated by bubbling N_2_ into the solution for 10 min. During the reaction, gas samples (50 μL) were taken at different time intervals using a gastight syringe and quantified by gas chromatography in a Shimadzu GC-2010 Plus equipped with a ShinCarbon ST column (2 m × 2 mm i.d.) and a barrier discharge ionization detector (BID). Photocatalytic tests were performed independently in quintuplicate, and results were expressed as mean values with standard deviations.

## Supporting Information Available

Composition of LDH and composites; XRD patterns of graphite and graphene oxide; Thermogravimetric analysis curves for LDH synthesized materials; Raman spectra of all synthesized materials; FTIR-ATR spectra of the synthesized materials; N_2_ adsorption–desorption isotherm of GO and LDH-based materials; SEM images of LDH-based materials; TEM images of GO, GQD, LDHp, and LDHu; Particle size distributions of GQD and LDH-based materials by TEM; UV–vis absorption spectra and band gap representation; Reusability experiments of LDHu-GO and LDHu-GQD; XRD pattern and FTIR-ATR spectra of LDHu-GO and LDHu-GQD after 72 h of irradiation; Photoluminescence (PL) measurements; Langmuir isotherm of Ru(bpy)_3_^2+^ adsorption on GO (PDF)
