# Supplementary material for: Visible-Light-Driven Photocatalytic H2 Production Using Composites of Co–Al Layered Double Hydroxides and Graphene Derivatives
Source: Inorg Chem. 2024 May 28;63(23):10500–10. doi: 10.1021/acs.inorgchem.4c00671 (PMC11167638; doi:10.1021/acs.inorgchem.4c00671)
Supplement: Supplementary file 1 — ic4c00671_si_001.pdf [file ic4c00671_si_001.pdf]

Supporting information

Visible-light-driven photocatalytic H_2 production using composites of Co-Al layered double hydroxides and graphene derivatives

Dolores G. Gil-Gavilán[a], Juan Amaro-Gahete[a,b], Daniel Cosano[a], Miguel Castillo-Rodríguez[c], Gustavo de Miguel[d], Dolores Esquivel[a], José R. Ruiz[a], Francisco J. Romero-Salguero[a*]*

[a]Departamento de Química Orgánica, Instituto Químico para la Energía y el Medioambiente (IQUEMA), Facultad de Ciencias, Universidad de Córdoba, Campus de Rabanales, Edificio Marie Curie, 14071 Córdoba, Spain.

[b]UGR-Carbon – Materiales Polifuncionales Basados en Carbono, Departamento de Química Inorgánica, Unidad de Excelencia Química Aplicada a Biomedicina y Medioambiente, Universidad de Granada, 18071 Granada, Spain.

[c]Departamento de Física Aplicada, Radiología y Medicina Física, Universidad de Córdoba, Campus de Rabanales, 14071 Córdoba, Spain.

[d]Departamento de Química Física y Termodinámica Aplicada, Instituto Químico para la Energía y el Medioambiente (IQUEMA), Facultad de Ciencias, Universidad de Córdoba, Campus de Rabanales, Edificio Marie Curie, 14071 Córdoba, Spain.

* Corresponding authors: Francisco J. Romero-Salguero (qo2rosaf@uco.es), José R. Ruiz (qo1ruarj@uco.es)

Table S1. Composition of LDH and composites.

Material	Co (mmol g^{-1})[a]	Al (mmol g^{-1})[a]	Co/Al molar ratio[a]	Carbon (wt%)[b]	H_2O (mmol g^{-1})[c]
**LDHp**	6.7	2.1	3.2		4.9
**LDHp-GO**	6.3	1.9	3.3	5.5	5.8
**LDHp-GQD**	6.5	2.0	3.3	5.6	6.2
**LDHu**	6.8	2.2	3.1		6.4
**LDHu-GO**	6.2	2.0	3.1	6.6	6.6
**LDHu-GQD**	6.1	2.0	3.1	12.3	7.6

[a]Based on XRF measurements; [b]Determined by elemental analysis; [c]Determined by ATG analyses.

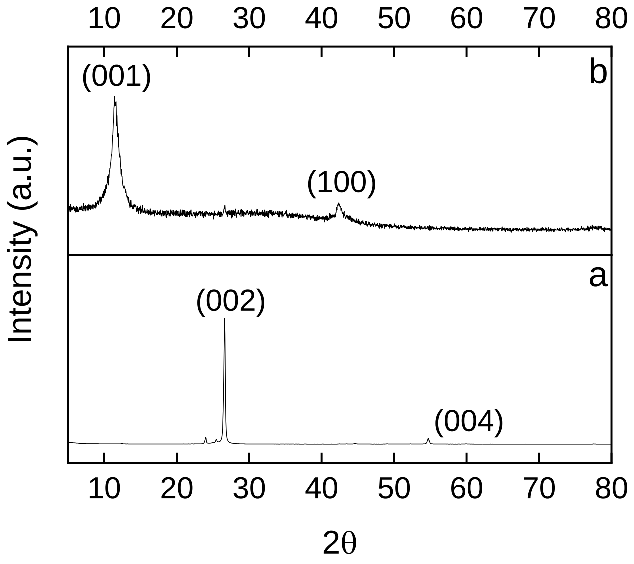

Figure S1. XRD patterns of (a) graphite and (b) graphene oxide (GO).

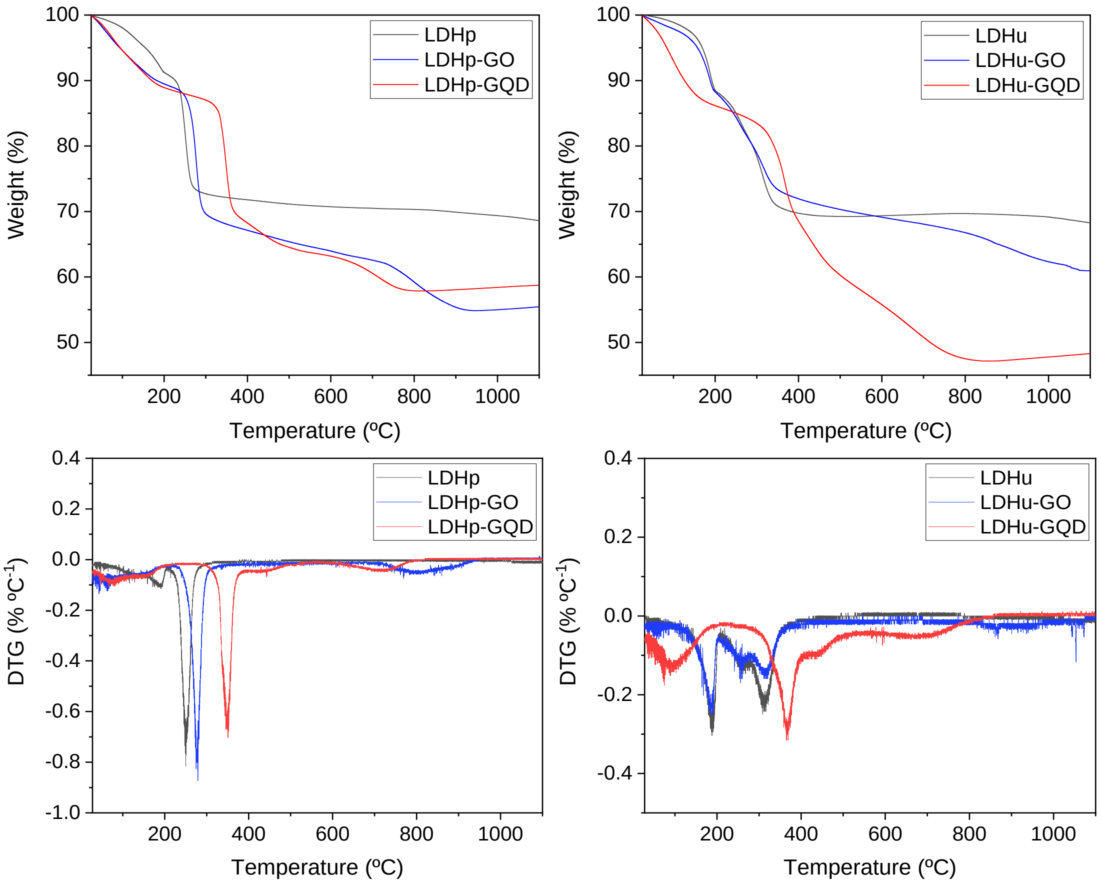

Figure S2. Thermogravimetric analysis curves for LDH synthesized materials.

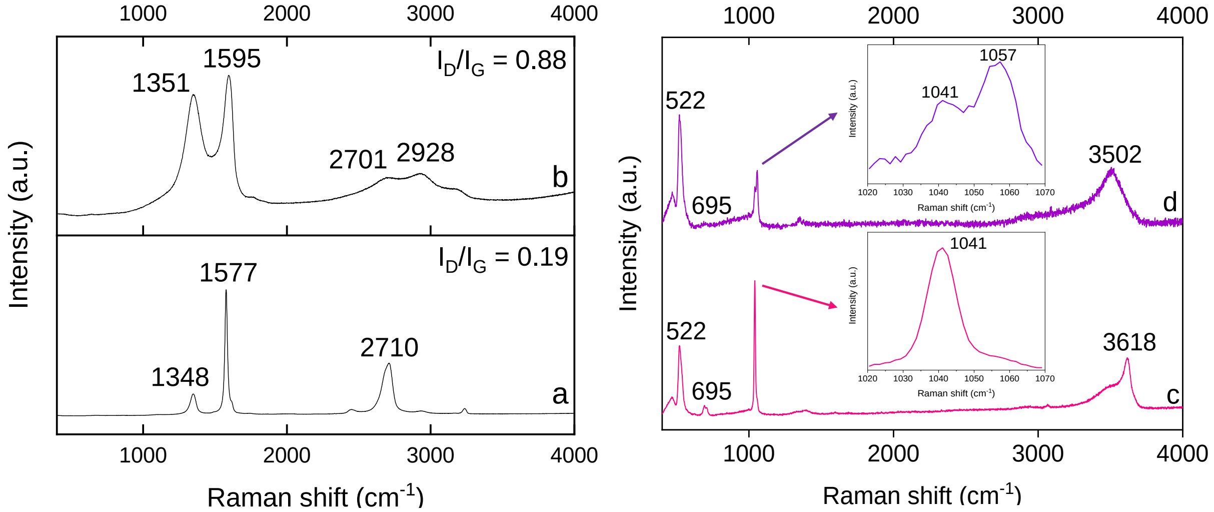

Figure S3. Raman spectra of (a) graphite, (b) GO, (c) LDHp and (d) LDHu.

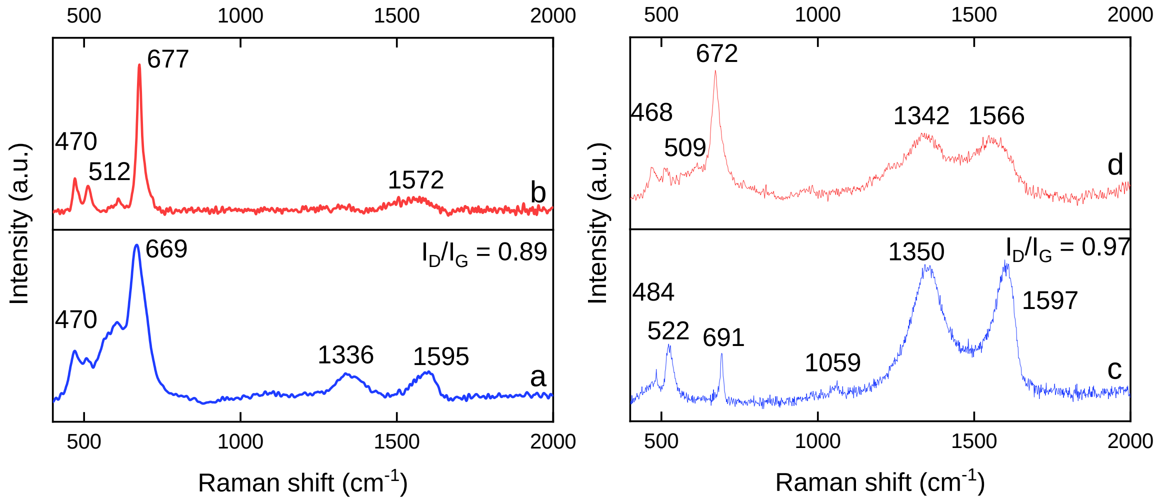

Figure S4. Raman spectra of synthesized composites: (a) LDHp-GO, (b) LDHp-GQD, (c) LDHu-GO and (d) LDHu-GQD.

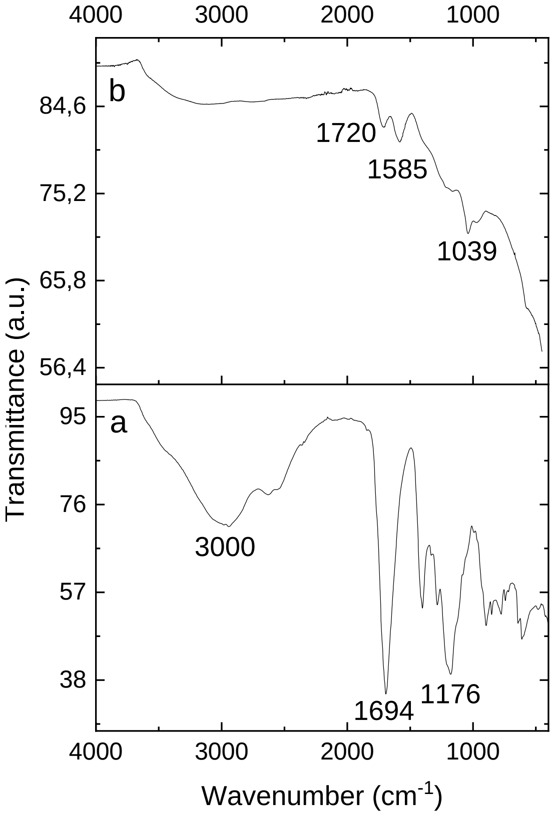

Figure S5. FTIR-ATR spectra of (a) GQD and (b) GO. The GQD spectrum revealed a broad band at 3000 cm^{-1} associated with O-H stretching of hydroxyl groups in carboxylic acid groups, which was corroborated by signals at 1694 and 1176 cm^{-1} assigned to C=O and C-O bonds, respectively, due to incomplete carbonization of citric acid during the synthesis and so the presence of oxygenated functionalities in the GQD [1]. In GO spectrum, the main peaks were associated to carbonyl C=O stretching at 1720 cm^{-1}, aromatic C=C stretching at 1585 cm^{-1} and C-O stretching at 1039 cm^{-1} [2].

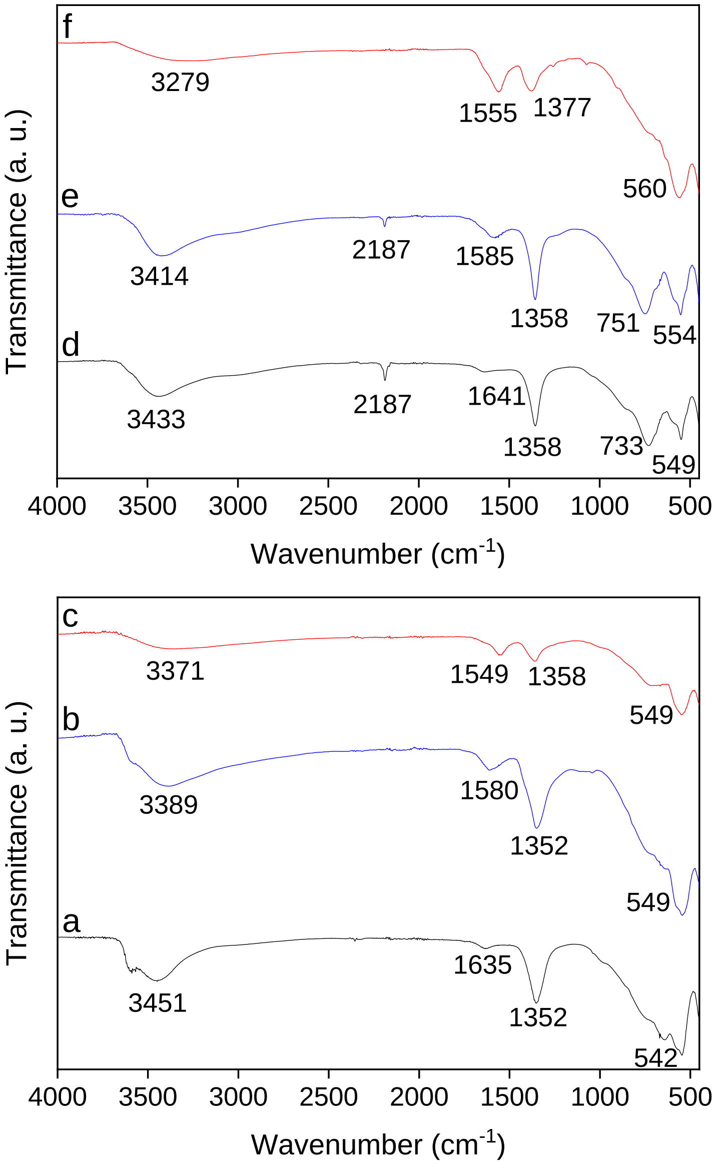

Figure S6. FTIR-ATR spectra of composites: (a) LDHp, (b) LDHp-GO, (c) LDHp-GQD, (d) LDHu, (e) LDHu-GO and (f) LDHu-GQD.

FTIR-ATR assignments:

FTIR-ATR spectra corroborated the formation of composites, according to the bands observed at ca. 3500 cm^{-1}, attributed to OH stretching, at 2187 cm^{-1}, which corresponded to cyanate groups (CNO^{-}), present in intermediate decomposition products from the hydrolysis of urea [3], at approximately 1620 cm^{-1}, assigned to adsorbed water, and at 1550-1585 cm^{-1}, ascribed to C=C stretching vibrations. Those bands at 1352-1377 cm^{-1} were associated to the stretching mode of both NO_3^{-} and CO_3^{2-} present in LDH layers[4]. Bands below 800 cm^{-1} were assigned to metal-oxygen (M-O) stretching and bending vibrations in the brucite-like lattice [5].

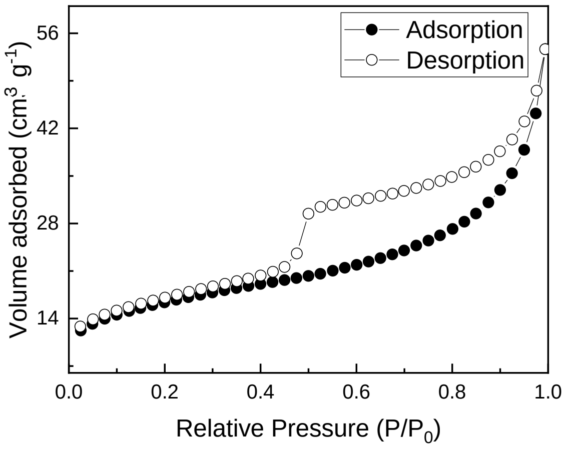

Figure S7. N_2 adsorption-desorption isotherm of GO. GO presented a type IV isotherm with hysteresis loop at a relative pressure between 0.45 and 0.96, specific surface area of 59 m² g^{-1} and pore volume of 0.066 cm^3 g^{-1}.

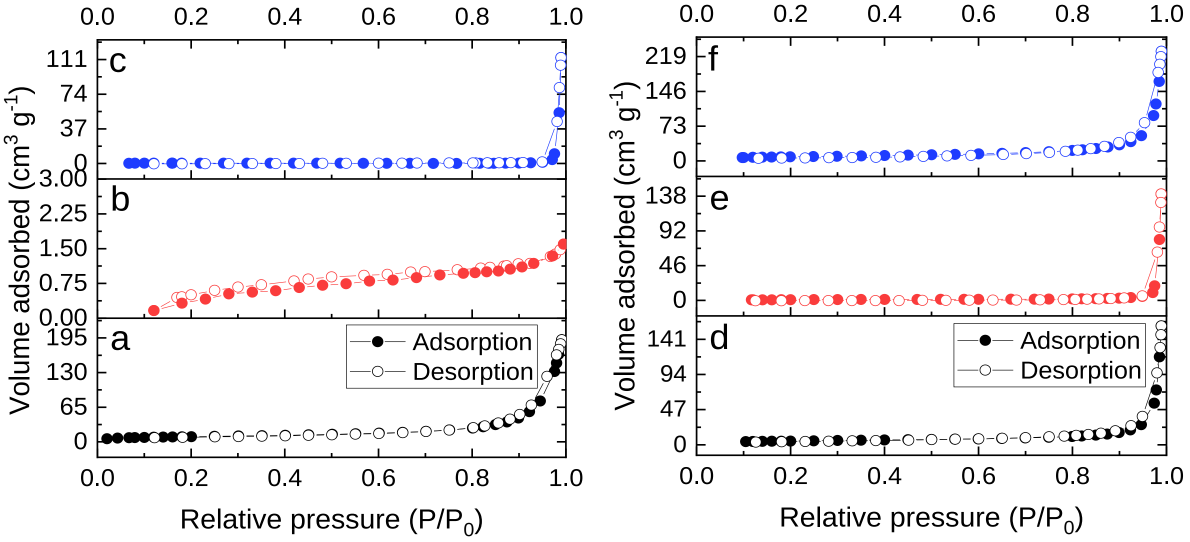

Figure S8. N_2 adsorption-desorption isotherms of all synthesized materials. Left: co-precipitation obtained composites: a) LDHp, b) LDHp-GQD and c) LDHp-GO. Right: urea hydrolysis-obtained composites: d) LDHu, e) LDHu-GQD and f) LDHu-GO.

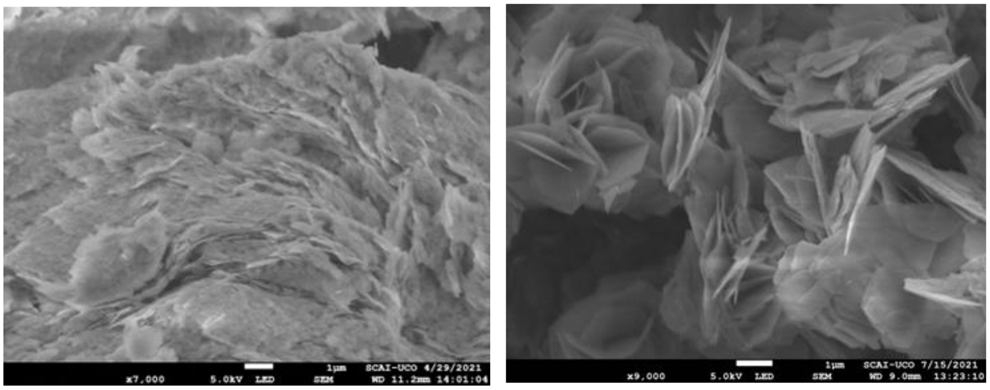

Figure S9. SEM images of pristine LDH materials. LDHp (left) and LDHu (right).

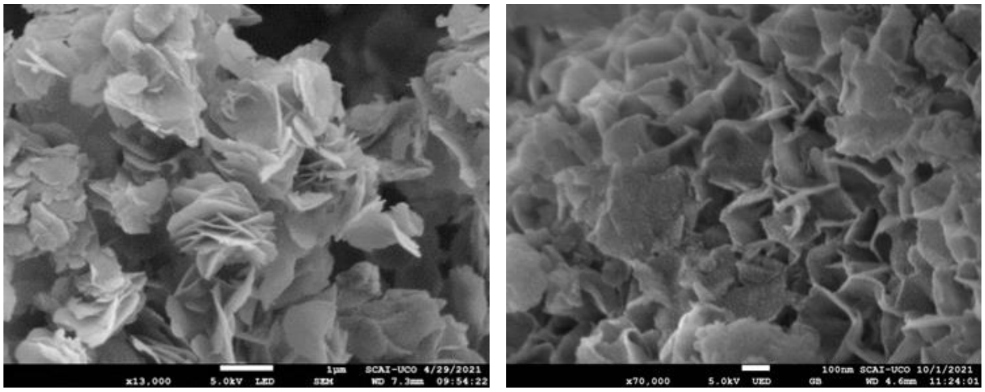

Figure S10. SEM images of urea hydrolysis obtained composites. LDHu-GO (left) and LDHu-GQD (right).

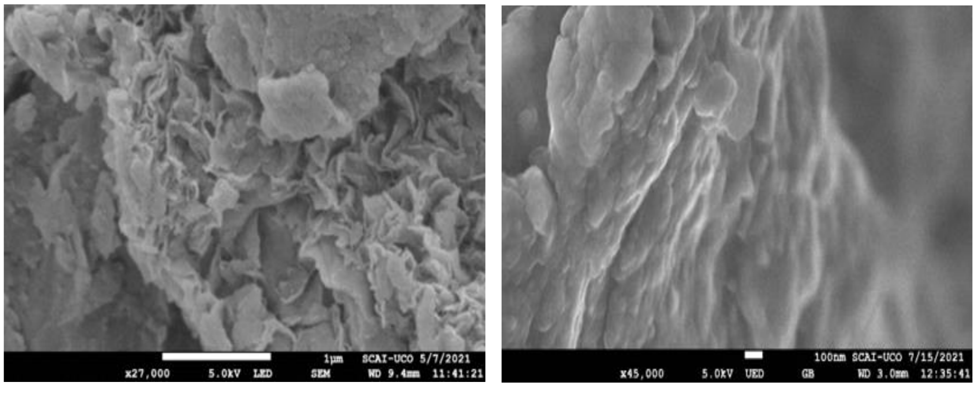

Figure S11. SEM images of co-precipitation obtained composites. LDHp-GO (top) and LDHp-GQD (bottom).

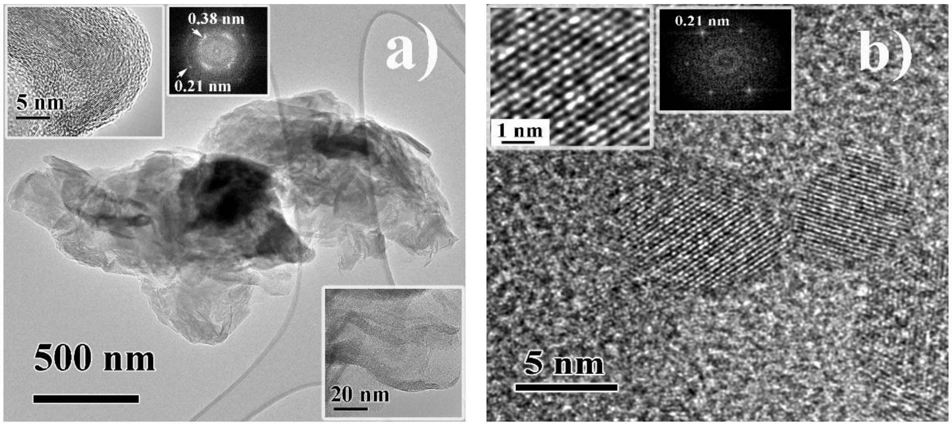

Figure S12. a) Graphene oxide with two insets at higher magnification. Fast Fourier Transform (FFT) analysis provides typical interplanar distances for GO. b) Graphene Quantum Dots (GQD). One of them is magnified at the upper left inset whose corresponding FFT is displaying (100) planes with a spacing of 0.21 nm.

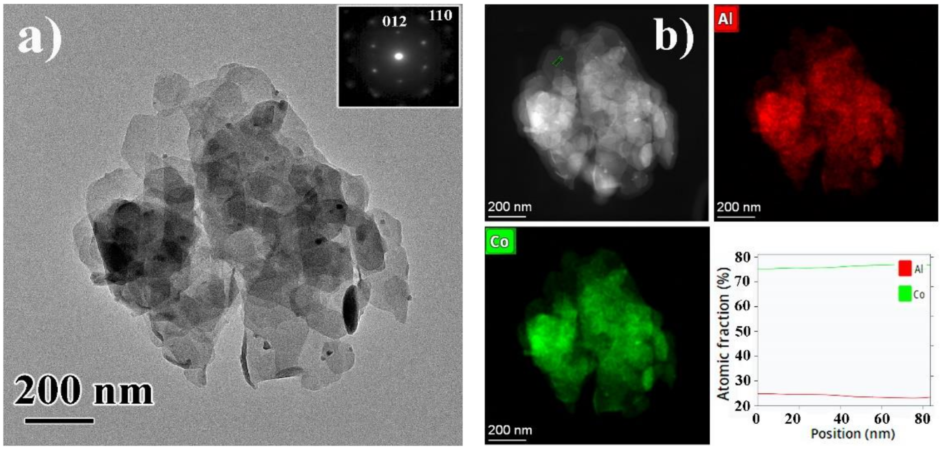

Figure S13. a) TEM micrograph of pristine LDH synthetized by co-precipitation (LDHp). b) HAADF and Al and Co elemental maps are shown. Atomic % profile confirmed a 3:1 Co:Al ratio.

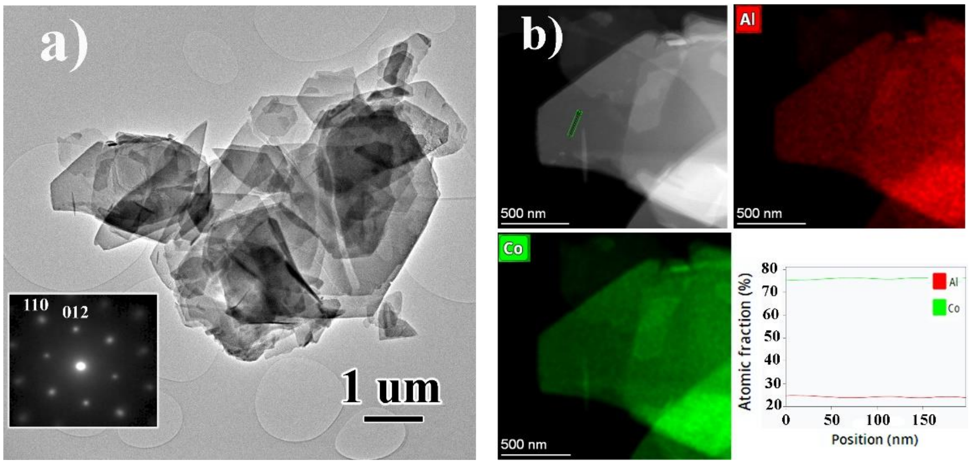

Figure S14. a) TEM micrograph of pristine LDH synthetized by urea homogeneous precipitation (LDHu). b) HAADF and Al and Co elemental maps are shown. Again, a 3:1 (Co:Al) ratio was obtained.

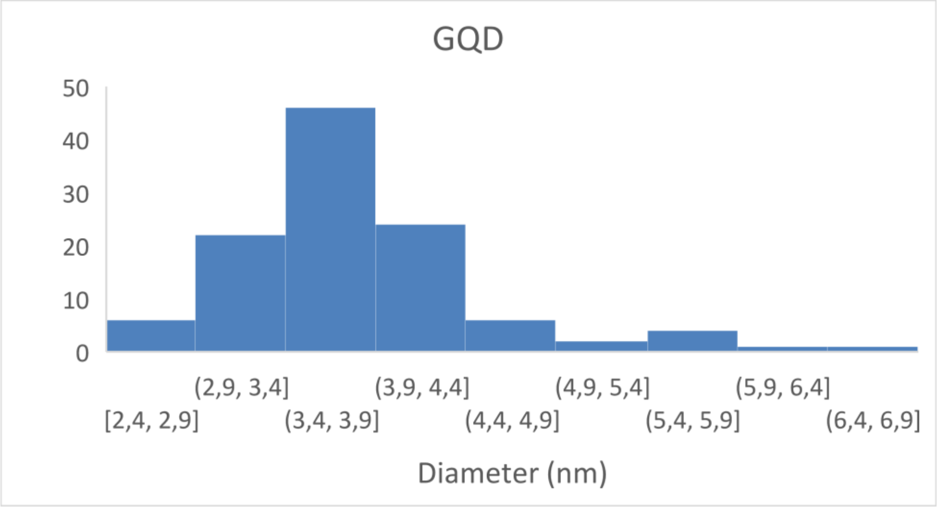

Figure S15. Particle size distribution of GQD by TEM.

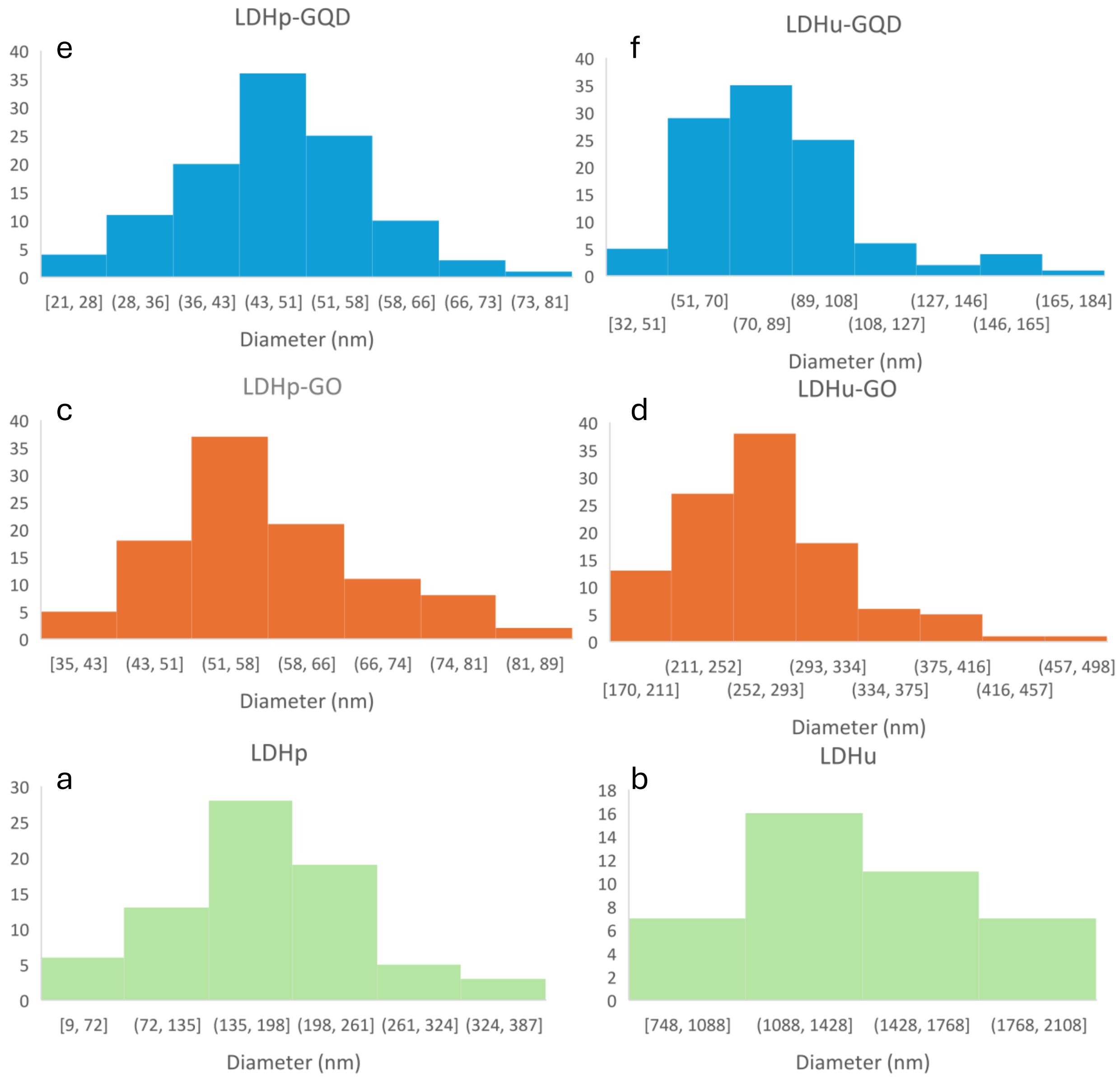

Figure S16. Particle size distribution of (a) LDHp, (b) LDHu, (c) LDHp-GO, (d) LDHu-GO, (e)LDHp-GQD and (f) LDHu-GQD by TEM.

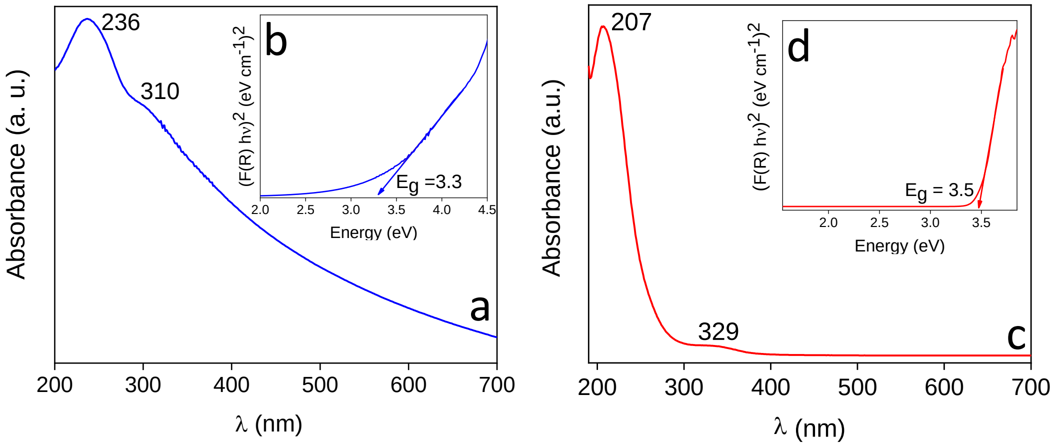

Figure S17. UV-vis absorption spectra of a) GO and c) GQD. Band gap of b) GO and d) GQD. GO presented an absorption band at 236 nm attributed to the π → π* transition of aromatic rings and a shoulder at 310 nm corresponding to the n → π* transition of C=O bonds [6]. Bands at 207 nm and 329 nm attributed to π → π* and n → π* transitions, respectively, were present in GQD [7]. The band gap for GO and GQD was 3.3 eV and 3.5 eV, respectively.

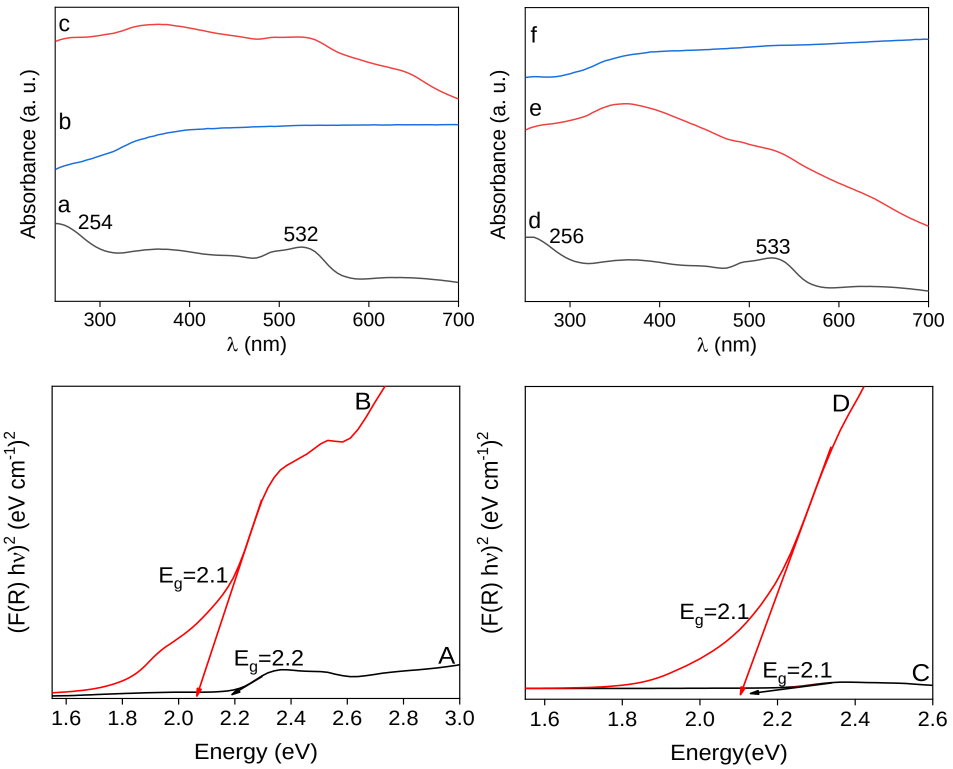

Figure S18. UV-vis diffuse reflectance spectra (top) and materials band gap (bottom): (a) LDHp, (b) LDHp-GO, (c) LDHp-GQD, (d) LDHu, (e) LDHu-GO and (f) LDHu-GQD. Band gaps of A) LDHp, B) LDHp-GQD, C) LDHu and D) LDHu-GQD.

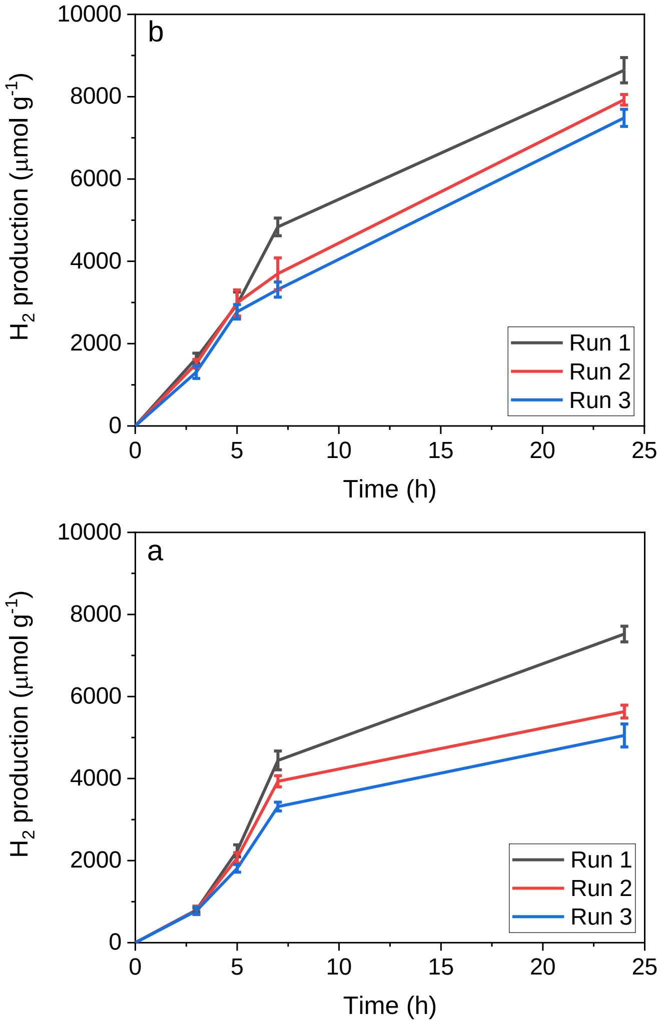

Figure S19. Reusability experiments of a) LDHu-GO and b) LDHu-GQD.

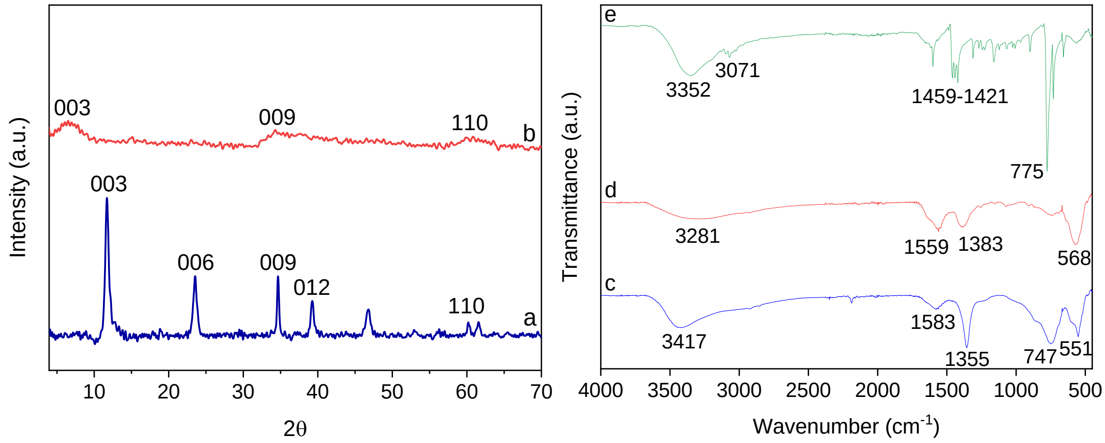

Figure S20. Characterization of composites after 72 h of irradiation. Left: XRD patterns of (a) LDHu-GO and (b) LDHu-GQD. Right: FTIR-ATR spectra of (c) LDHu-GO, (d) LDHu-GQD and (e) $Ru(bpy)_3^{2+}$, for comparison.

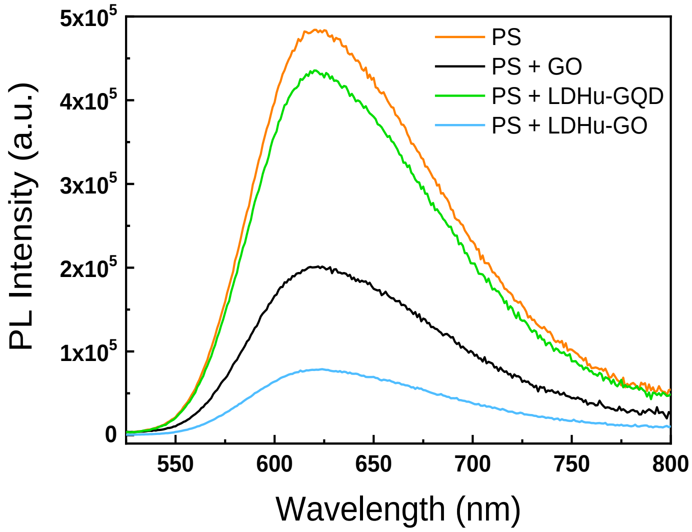

Figure S21. Photoluminescence (PL) measurements.

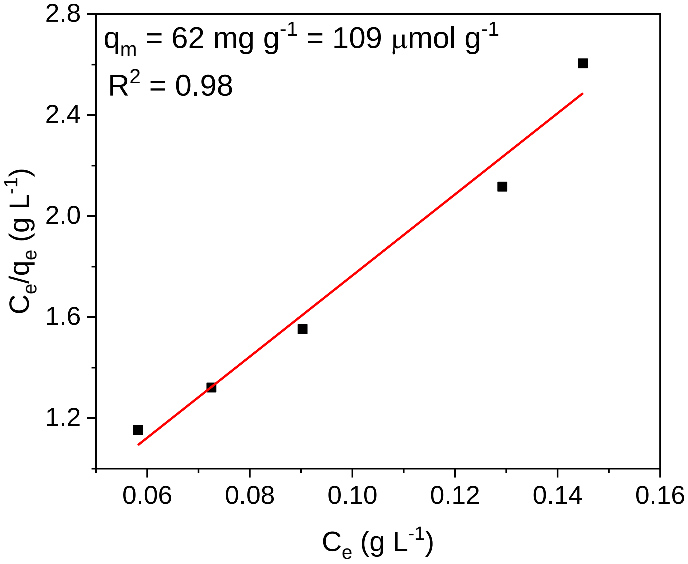

Figure S22. Langmuir isotherm of $Ru(bpy)_3^{2+}$ adsorption on GO.

REFERENCES

(1) Sarabiyan Nejad, S.; Babaie, A.; Bagheri, M.; Rezaei, M.; Abbasi, F.; Shomali, A. Effects of Graphene Quantum Dot (GQD) on Photoluminescence, Mechanical, Thermal and Shape Memory Properties of Thermoplastic Polyurethane Nanocomposites. *Polym. Adv. Technol.* **2020**, *31* (10), 2279–2289. https://doi.org/10.1002/pat.4948.

(2) Stylianakis, M. M.; Kosmidis, D. M.; Anagnostou, K.; Polyzoidis, C.; Krassas, M.; Kenanakis, G.; Viskadouros, G.; Kornilios, N.; Petridis, K.; Kymakis, E. Emphasizing the Operational Role of a Novel Graphene-Based Ink into High Performance Ternary Organic Solar Cells. *Nanomaterials* **2020**, *10* (1), 89. https://doi.org/10.3390/nano10010089.

(3) Zhang, Y. C. Hydrothermal Synthesis of Ni-Fe Layered Double Hydroxide with High Crystallinity Using Homogeneous Precipitation Method. *Adv. Mater. Res.* **2013**, *821–822*, 1313–1316. https://doi.org/10.4028/www.scientific.net/AMR.821-822.1313.

(4) Pálinkó, I.; Sipos, P.; Berkesi, O.; Varga, G. Distinguishing Anionic Species That Are Intercalated in Layered Double Hydroxides from Those Bound to Their Surface: A Comparative IR Study. *J. Phys. Chem. C* **2022**, *126* (36), 15254–15262. https://doi.org/10.1021/acs.jpcc.2c03547.

(5) Baruah, A.; Mondal, S.; Sahoo, L.; Gautam, U. K. Ni-Fe-Layered Double Hydroxide/N-Doped Graphene Oxide Nanocomposite for the Highly Efficient Removal of Pb(II) and Cd(II) Ions from Water. *J. Solid State Chem.* **2019**, *280*, 120963. https://doi.org/10.1016/j.jssc.2019.120963.

(6) Yang, Y.; Liu, T. Fabrication and Characterization of Graphene Oxide/Zinc Oxide Nanorods Hybrid. *Appl. Surf. Sci.* **2011**, *257* (21), 8950–8954. https://doi.org/10.1016/j.apsusc.2011.05.070.

(7) Dong, Y.; Shao, J.; Chen, C.; Li, H.; Wang, R.; Chi, Y.; Lin, X.; Chen, G. Blue Luminescent Graphene Quantum Dots and Graphene Oxide Prepared by Tuning the Carbonization Degree of Citric Acid. *Carbon N. Y.* **2012**, *50* (12), 4738–4743. https://doi.org/10.1016/j.carbon.2012.06.002.
